# ESO Guideline on covert cerebral small vessel disease

**DOI:** 10.1177/23969873211012132

**Published:** 2021-05-11

**Authors:** Joanna M Wardlaw, Stephanie Debette, Hanna Jokinen, Frank-Erik De Leeuw, Leonardo Pantoni, Hugues Chabriat, Julie Staals, Fergus Doubal, Salvatore Rudilosso, Sebastian Eppinger, Sabrina Schilling, Raffaele Ornello, Christian Enzinger, Charlotte Cordonnier, Martin Taylor-Rowan, Arne G Lindgren

**Affiliations:** 1 Centre for Clinical Brain Sciences, UK Dementia Research Institute, University of Edinburgh, Edinburgh, UK; 2 Bordeaux Population Health Center, University of Bordeaux, INSERM, UM1219, Team VINTAGE; 3 Department of Neurology, Institute for Neurodegenerative Disease, Bordeaux University Hospital, Bordeaux, France; 4 HUS Neurocenter, Division of Neuropsychology, Helsinki University Hospital, University of Helsinki and Department of Psychology and Logopedics, Faculty of Medicine, University of Helsinki, Finland; 5 Radboud University Medical Center, Department of Neurology; Donders Center for Medical Neuroscience, Nijmegen, The Netherlands; 6 Stroke and Dementia Lab, 'Luigi Sacco' Department of Biomedical and Clinical Sciences, University of Milan, Milano, Italy; 7 Department of Neurology, Hopital Lariboisiere, APHP, INSERM U 1161, FHU NeuroVasc, University of Paris, Paris, France; 8 Department of Neurology, School for Cardiovascular Diseases (CARIM), Maastricht UMC+, AZ Maastricht, the Netherlands; 9 Dept of Medicine for the Elderly, University of Edinburgh, Edinburgh, UK; 10 Comprehensive Stroke Center, Department of Neuroscience, Hospital Clínic, Barcelona, Spain; 11 University Clinic of Neurology, Medical University of Graz, Graz, Austria; 12 Department of Applied Clinical Sciences and Biotechnology, University of L’Aquila, L’Aquila, Italy; 13 Univ. Lille, INSERM, CHU Lille, U1172, LilNCog – Lille Neuroscience & Cognition, Lille, France; 14 Institute of Cardiovascular and Medical Sciences, University of Glasgow, Glasgow, UK; 15 Department of Clinical Sciences Lund, Neurology, Lund University; Section of Neurology, Skåne University Hospital, Lund, Sweden

**Keywords:** White matter hyperintensities, lacunes, cerebral small vessel disease, leukoaraiosis, silent brain infarcts, covert, prevention, stroke, dementia, recommendations, outcome

## Abstract

‘Covert’ cerebral small vessel disease (ccSVD) is common on neuroimaging in persons without overt neurological manifestations, and increases the risk of future stroke, cognitive impairment, dependency, and death. These European Stroke Organisation (ESO) guidelines provide evidence-based recommendations to assist with clinical decisions about management of ccSVD, specifically white matter hyperintensities and lacunes, to prevent adverse clinical outcomes. The guidelines were developed according to ESO standard operating procedures and Grading of Recommendations, Assessment, Development, and Evaluation (GRADE) methodology. We prioritised the clinical outcomes of stroke, cognitive decline or dementia, dependency, death, mobility and mood disorders, and interventions of blood pressure lowering, antiplatelet drugs, lipid lowering, lifestyle modifications, glucose lowering and conventional treatments for dementia. We systematically reviewed the literature, assessed the evidence, formulated evidence-based recommendations where feasible, and expert consensus statements. We found little direct evidence, mostly of low quality. We recommend patients with ccSVD and hypertension to have their blood pressure well controlled; lower blood pressure targets may reduce ccSVD progression. We do not recommend antiplatelet drugs such as aspirin in ccSVD. We found little evidence on lipid lowering in ccSVD. Smoking cessation is a health priority. We recommend regular exercise which may benefit cognition, and a healthy diet, good sleep habits, avoiding obesity and stress for general health reasons. In ccSVD, we found no evidence for glucose control in the absence of diabetes or for conventional Alzheimer dementia treatments. Randomised controlled trials with clinical endpoints are a priority for ccSVD.

## Introduction

Cerebral small vessel disease (SVD) refers to the presence of brain lesions found on CT or MR brain imaging or pathology examination, thought to have resulted from disease of the small blood vessels that perforate into the brain, primarily the white matter and deep grey matter. The full spectrum includes covert cerebral SVD (ccSVD) detected incidentally on neuroimaging, and SVD-related clinical presentations with stroke, cognitive decline or dementia, mood or physical dysfunction. The best described pathological-anatomical cerebral lesions are white matter hyperintensities (WMH), lacunes, microbleeds and enlarged perivascular spaces.^1^

SVD is considered to cause 25% of ischaemic strokes and most haemorrhagic strokes in older patients. SVD is also the commonest cause of vascular cognitive impairment and vascular dementia and is common in mixed dementias with other dementia pathologies, and is also a common cause of gait and balance problems and mood disorders in older people.^2^ Individuals with SVD may therefore present to stroke services, cognitive, movement disorder or neuropsychiatry clinics.^3^

However, findings suggesting SVD are also commonly found on CT or MRI brain imaging performed for investigation of other disorders in persons with no apparent neurological history, causing the lesions to be referred to as ‘silent’ or ‘covert’ cerebral SVD, henceforth in this Guideline referred to as ccSVD. Therefore, subjects with CT or MRI signs of ccSVD are often encountered by general practitioners and at various clinics. The number of persons with ccSVD lesions, and the amount and extent of ccSVD lesions per individual, increase with ageing,^4,5^ meaning that the number or volume of lesions that could be considered ‘normal’ varies with age.^4^ These lesions usually extend with time, although they may also recover.^6,7^ ccSVD is important, since it increases the risk of future stroke, dementia or death, both in persons with ccSVD lesions^5^ and in patients presenting with stroke of any type.^8^ Since increasing evidence indicates that the burden of MRI-markers of cSVD is associated with risk of complications at any age, and we do not know of any work identifying ‘safe’ thresholds of SVD, we prefer not to encourage use of ‘age norms’ at present.

We summarise here a few general comments which are relevant to the concepts behind this Guideline. SVD, with its covert, multiple clinical coincident expressions, and numerous outcomes of importance, does not easily lend itself to the conventional approaches required in formulating clinical guidelines. The topic is massive, difficult to identify, and relevant information may be buried in secondary publications or seemingly irrelevant studies. This theme thus presents complexities and practical barriers. On the basis of this, in this first part of the ESO ccSVD Guideline, our group reached a consensus to focus on the following areas: ccSVD primarily defined as WMH and lacunes (population, P); pharmacological interventions for stroke prevention (antihypertensive, antiplatelet, lipid lowering), lifestyle interventions for stroke or dementia prevention or healthy ageing (smoking cessation, weight reduction, dietary interventions, physical exercise, cognitive/social interventions, sleep/CPAP, or a mixture of these), glucose control, and conventional pharmacological anti-Alzheimer dementia treatments (interventions, I). The comparators are absence of the above intervention, or ‘best medical practice’, or less intense version of the intervention e.g. single vs dual antiplatelet agents, or target-based intervention such as blood pressure (BP) <140 mm Hg versus <120 mm Hg (comparator, C); and clinical outcomes are clinically apparent stroke, dependency, death, major adverse cardiac events (MACE), systemic adverse events like bleeding, cognitive decline, dementia, mobility including falls, and mood including depression (outcomes, O).

Although SVD lesion progression as observed on neuroimaging is a commonly reported research outcome of interest, our Group did not rate this highly enough to be included as an outcome (O) in this clinical guideline since robust clinical outcomes are of more importance to clinical services and to patients. However, in selected parts of this document, we comment briefly on imaging lesion changes.

Monogenic forms of ccSVD were not included since they were recently addressed in an EAN Guideline.^9^

Finally, a particular challenge was to define ‘ccSVD’. Increasing evidence indicates that SVD lesions are not ‘silent’ especially when present in larger numbers involving more areas of the brain. In fact patients with higher burdens of ccSVD lesions have atypical neurological, neuropsychiatric and cognitive symptoms (summarised in^10^) but these are poorly recognised in clinical practice and probably also by patients, and therefore have not received much attention and have not, as yet, been used to identify relevant patients in clinical trials. Furthermore, SVD affects multiple cognitive domains including memory and not just executive function as is commonly thought.^11,12^ At the present time, there is no formal clinical definition that distinguishes ccSVD which is truly silent from symptom-associated ccSVD. Therefore our Guideline Group adopted a pragmatic approach, focusing on patients with no formal diagnosis of TIA/stroke, cognitive impairment or dementia, mobility or mood disorders. However we recognise that ccSVD lesions are likely to be present in persons who are older, or have major risk factors such as hypertension or diabetes, even if their burden of SVD lesions has not been formally assessed, and that some ccSVD patients may have subtle undetected or unassessed changes in cognitive performance, mood or mobility.

## Methods

These guidelines were initiated by the ESO. A Module Working Group (MWG) was established, consisting of 11 experts (JMW, Chair; AGL, co-Chair; HC, CC, SD, F-EDL, FD, CE, HJ, LP, JS). The MWG was joined by four fellows during study screening (RO, SE, SR, SS) who assisted with abstract and full text screening and drafting the text. The MWG included eight neurologists, one neuropsychologist, one stroke physician, one neuroradiologist, all experts in SVD with interests in neuroepidemiology, cognitive testing, genetic, sporadic ischaemic and haemorrhagic SVDs. Fellows were all either trainee neurologists or post-doctoral fellows interested in stroke or early career epidemiologists. The composition of this group was approved by the ESO Guidelines Board and the ESO Executive Committee, based on a review of the intellectual and financial disclosures of the proposed members.

The guidelines were developed using Grading of Recommendations, Assessment, Development, and Evaluation (GRADE) methodology^13^ and the ESO Standard Operating Procedure,^14^ as described previously.^15^

The MWG developed a list of topics, and corresponding outcomes of clinical interest. The outcomes were rated as critical, important, or of limited importance according to GRADE criteria.^13,14^

## Selection of population, intervention, comparator, and outcome

Interventions in ccSVD might include those used for stroke or dementia primary or secondary prevention, lifestyle changes, antidepressant and other interventions and outcomes including worsening lesion burden, stroke, dementia, mobility and mood, dependency, major adverse cardiovascular events and death. The MWG voted in a closed ballot to identify which PICO questions were considered to be the highest priority. Considering the full range of clinical presentations in SVD and constructing the complete PICO questions initially resulted in approximately 36 PICO questions which were clearly unworkable with the time and resources available. The MWG decided to prioritize this first ESO SVD Guideline on patients with ccSVD in whom no guidelines are currently available, above patients presenting with stroke, cognitive decline, mobility or mood disorders whose management is partly covered by broader guidelines on these conditions. The MWG selected three stroke prevention treatments (BP lowering, antiplatelet drugs, lipid lowering), lifestyle interventions (smoking cessation, exercise, diet including vitamins, cognitive behavior therapy), glucose lowering agents and anti-dementia treatments as key Interventions for which the effect in ccSVD was uncertain. The MWG prioritized the absence or a lesser amount of the intervention as Comparators (for example, BP or lipid or glucose lowering might be compared to less intense lowering of these parameters or to avoiding the lowering intervention altogether – both options were considered), and chose clinical outcomes of stroke (ischaemic or haemorrhagic), cognitive decline or dementia, MACE, death, dependency, loss of mobility, and mood disorders as clinically important Outcomes. Non-clinical outcomes such as blood biomarkers, WMH progression or silent infarcts were excluded from the PICO questions for aforementioned reasons.

The MWG focused on WMH and lacunes as the ccSVD lesions of interest. We did not examine two other subtypes of ccSVD: microbleeds where there are recent reviews and large studies^16^ or enlarged perivascular spaces because their definition and clinical importance are more unclear and studies still scarce.

The MWG formulated six main PICO (Population, Intervention, Comparator, Outcome) questions each with several sub-questions relating to the seven different Outcomes of interest. These were subsequently approved by the ESO Guidelines board and the ESO Executive Committee.

For each PICO question, search terms were identified, tested, refined and agreed by JMW and AGL with the ESO Guidelines methodologist, Martin Taylor-Rowan (MT-R). Search terms are listed in the **Supplementary materials**.

## Identification and selection relevant studies

MT-R then conducted systematic searches of the PubMed, Embase, and Cochrane Library databases, covering the period from the inception of each database to 11th Dec 2020. The search strategy was informed by terminology for SVD identified in other SVD research^1^ and for each intervention and outcome. Strategies were tested and modified to optimise sensitivity and specificity before the final search was run. The search results were loaded into the web-based platform Covidence for assessment and consequent systematic review by the MWG.

Different combinations of two MWG authors independently screened the titles and abstracts of publications registered in Covidence and assessed the full text of potentially relevant studies. We focused on randomized controlled trials and systematic reviews of RCTs, but also considered other types of study such as health registry data analyses, large observational studies (minimum size 100 subjects) and systematic reviews or individual patient data meta-analyses of observational studies since we anticipated a lack of high quality RCTs. We noted potentially relevant ongoing studies for future reference. All disagreements were resolved by discussion between the two authors or by a third MWG author. We also searched reference lists of review articles, the authors own reference libraries, and previous guidelines for additional relevant material.

For each question, a group of three or four MWG members assisted by one or more Fellows (a ‘PICO group’, details see author contributions) was formed to evaluate the available evidence. The risk of selection, performance, detection, attrition and reporting biases in each randomised trial was assessed using the Cochrane Collaboration’s tool,^17^ and heterogeneity across studies was assessed using Cochran’s Q (reported as a p value) and I^2^ statistics.^18^

For each PICO question and each outcome, the quality of evidence was rated using the GRADEpro Guideline Development Tool (McMaster University, 2015; developed by Evidence Prime, Inc.) using guidelines for non-pooled data as necessary,^19^ as high, moderate, low or very low^14^ by MT-R and agreed by at least two members of each PICO group.

The relevant PICO group analysed the available primary and any additional data, prepared tables and figures, and drafted three sections of text: ‘analysis of current evidence’ which focused on relevant RCTs and/or systematic reviews; ‘additional information’ to summarise indirect evidence and provide context about the Intervention of interest in ccSVD or related presentations; and ‘expert consensus statement’ if the PICO group considered that not enough evidence was available to provide an evidence-based recommendation for situations in which practical guidance is often needed in everyday clinical practice. Since there were few RCTs with clinical outcomes, where reasonable, we also describe effects of the Intervention of interest on WMH change and performed a meta-analysis where feasible using weighted random effects meta-analysis (relative weights for each study are based on relative sample size) on the standard mean difference of SVD change between the intervention vs control groups using ’Comprehensive Meta-analysis’ software (Version 3,

Borenstein M., Biostat, Englewood, NJ 2013). We assessed heterogeneity using the I^2^ statistic. This was possible for BP lowering and lipid lowering Interventions.

Each PICO Group formulated an evidence-based recommendation according to the GRADE evidence profiles and the ESO standard operating procedure.^14^

The Expert Consensus Statements are based on voting by all expert MWG members. Importantly, these Expert Consensus Statements should not be regarded as evidence-based recommendations, since they only reflect the opinion of the MWG.

The Guidelines document was reviewed several times by all MWG members, and modified using a Delphi approach until consensus was reached. The document was subsequently reviewed and approved by two external reviewers, members of the ESO Guidelines Board and Executive Committee, and the Editor of the *European Stroke Journal*.

## Results

**PICO 1:** In patients with covert cerebral small vessel diseases [WMH and/or lacunes], does antihypertensive treatment, compared to less intense or avoiding this intervention, reduce ischaemic or haemorrhagic strokes (1.1), cognitive decline or dementia (1.2), dependency (1.3), death (1.4), MACE (1.5), mobility (1.6), or mood disorders (1.7).

## Analysis of current evidence

**Figure 1.**
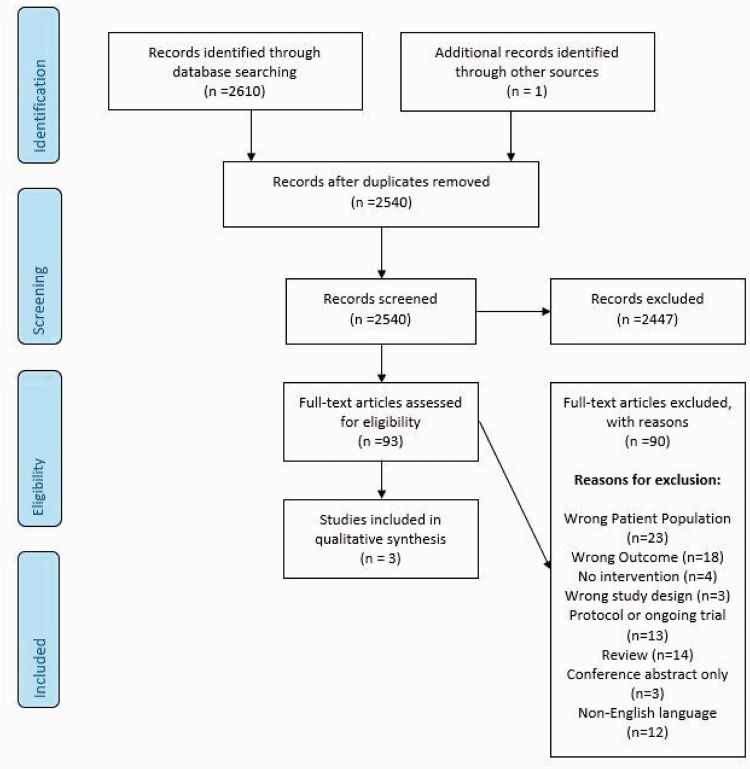
Search results PICO 1.

The literature search identified three trials addressing this PICO question (Figure 1).^20–22^ All three studies had a high risk of bias and suffered from imprecision due to very small sample size, as well as indirectness, due either to no control group for comparison,^22^ or the control group also being given intervention.^20,21^ Pooling of these trials was not possible, therefore we describe each and the outcomes that are addressed. Table 1 summarizes the findings for all PICO1 trials and Table 2 the quality of the results.

**Table 1.** Summary of clinical trial findings relevant for PICO1.

Outcome	Study	Study design	Population	Mean Age	Intervention	Comparison	Duration of follow-up	Outcome	IPD Intervention		IPD control		OR/HR/RR/Summary of findings	RoB rating
									N event	N Total	N event	N Total		
Stroke (ischaemic or haemorrhagic)	INFINITY	RCT	Age≥75 yrs, systolic HTN, WMH ≥0.5%	80.5 years	Intensive treatment^a^ (24 hr SBP ≤130 mmHg)	Standard Treatment^a^ (24 hr SBP≤145 mmHg)	3 years	Stroke	1	78	2	83	OR: 0.526 (95%CI=0.047-5.919)^b^	High
Cognitive decline or dementia	INFINITY	RCT	Age≥75 yrs, systolic HTN, WMH ≥0.5%	80.5 years	Intensive treatment^a^ (24 hr SBP ≤130 mmHg)	Standard Treatment^a^ (24 hr SBP≤145 mmHg)	3 years	Change in performance on neuropsychological tests	NA	78	NA	83	No significant difference, **except slower package sequential reaction time in intensive vs standard group, p<0.01)**^c^	High
	Pantoni 1996	Trial without controls	dementia, leukoaraiosis on CT (Hachinski ischaemic score >7)	62.9 years	Nimodipine	None	12 months	Neuropsychological tests; Blessed dementia scale	NA	31	NA	NA	**Improvement in scores start vs end for Corsi bock tapping test: (2.0+/-1.2 vs 2.5 +/-1.6, p=0.018)**; Other scores stable	High
	Zhang 2019	RCT	Age≥60 yrs, HTN	70.7 years	Telmisartan^d^	Placebo^d^	63 months	Cognitive impairment (MMSE&DRS defined)	59	366	48	366	OR: 0.758 (0.499–1.152) HR: 0.758 (0.518–1.114)^e^	High
Dependency	Pantoni 1996	Trial without controls	dementia, leukoaraiosis on CT (Hachinski ischaemic score >7)	62.9 years	Nimodipine	None	12 months	Akhtar Disability rating scale score	NA	31	NA	NA	Remained stable (no significance values reported); difference in scores start vs end 9.2+/-3.3 at entry vs 9.4 +/-4.8 at end	High
Death	INFINITY	RCT	Age≥75 yrs, systolic HTN, WMH ≥0.5%	80.5 years	Intensive treatment^a^ (24 hr SBP ≤130 mmHg)	Standard Treatment^a^ (24 hr SBP≤145 mmHg)	3 years	Deaths	2	78	4	83	OR: 0.520 (95%CI=0.092-2.921)^b^	High
MACE	INFINITY	RCT	Age≥75 yrs, systolic HTN, WMH ≥0.5%	80.5 years	Intensive treatment^a^ (24 hr SBP ≤130 mmHg)	Standard Treatment^a^ (24 hr SBP≤145 mmHg)	3 years	Serious non fatal cardiovascular events	4	78	17	83	**Risk Ratio: 0.24 (95% CI, 0.08–0.68)**	High
Mobility impairment	INFINITY	RCT	Age≥75 yrs, systolic HTN, WMH ≥0.5%	80.5 years	Intensive treatment^a^ (24 hr SBP ≤130 mmHg)	Standard Treatment^a^ (24 hr SBP≤145 mmHg)	3 years	Various mobility measurements	NA	78	NA	83	No significant change in gait speed (in sec): 0.04 (95%CI= −0.68∼0.76), p=0.91; similar for other mobility measures	High
Mood disorder	Pantoni 1996	Trial without controls	dementia, leukoaraiosis on CT (Hachinski ischaemic score >7)	62.9 years	Nimodipine	None	12 months	Hamilton depression rating scale score	NA	31	NA	NA	**Significant improvement during treatment course; Hamilton DRS: 13.3 +/-5.6 at beginning vs 9.7 +/- 5.9 at end; p<0.001**	High

Notes: Significant results are in bold.

RoB: risk of bias; OR: odds ratio; HR: hazard ratio; RR: risk ratio; IPD: individual patient data; HTN: hypertension; WMH: white matter hyperintensities; SBP: systolic blood pressure; CI: confidence interval; MMSE: mini mental state examination; DRS: dementia rating scale.

^a^Intensive treatment: lisinopril (or losartan if intolerance to lisinopril) and amlodipine, and if 3 drugs did not result in the goal SBP, other drugs, including β-blockers, aldosterone antagonists, or α-1 adrenergic blockers, could be added as fourth and fifth agents; standard treatment: lisinopril or losartan, depending on clinical history, and if SBP remained above goal, amlodipine was added.

^b^Calculated based on IPD data, unadjusted OR.

^c^Difference no longer significant when restricting to patients who reached goal BP and remained within their treatment group for the entire trial.

^d^An open-label medication, hydrochlorothiazide, was used as a baseline medication in all treatment arms.

^e^Adjusted for age, sex, education, smoking and alcohol consumption.

**Table 2.** Quality of results in trials relevant for PICO1.

Certainty assessment							№ of patients		Effect		Certainty	Importance
№ of studies	Study design	Risk of bias	Inconsistency	Indirectness	Imprecision	Other considerations	Antihypertensive treatment	No antihypertensive treatment	Relative (95% CI)	Absolute (95% CI)		
**PICO 1.1 does antihypertensive treatment (I), compared to avoiding this intervention (C ) reduce risk of ischaemic or haemorrhagic strokes?**												
1	Randomised trial	Very serious	Not serious	Serious	Very serious	None	78	83	**Not pooled**	**Not pooled**	⨁ Very Low	Critical
Note: Downgraded 2 points for high RoB; downgraded 2 points for imprecision (very small sample size with wide confidence intervals); downgraded 1 point for indirectness (control group also given intervention).												
**PICO 1.2 does antihypertensive treatment (I), compared to avoiding this intervention (C) reduce risk of cognitive decline or dementia?**												
3	Randomised trial	Very serious	Not serious	Serious	Very serious	None	475	449	**Not pooled**	**Not pooled**	⨁ Very Low	Critical
Note: Downgraded 2 points for high RoB; downgraded 2 points for imprecision (very small sample size).												
**PICO 1.5 does antihypertensive treatment (I), compared to avoiding this intervention (C) reduce risk of MACE?**												
1	Randomised trial	Very Serious	Not serious	Very serous	Very serious	None	78	83	**Not pooled**	**Not pooled**	⨁ Very Low	Critical
Note: Downgraded 2 points for high RoB; downgraded 2 points for imprecision (very small sample size); downgraded 1 indirectness control group also given intervention and 1 study has no control group).												
**PICO 1.6 does antihypertensive treatment (I), compared to avoiding this intervention (C ) reduce risk of Mobility impairment?**												
1	Randomised trial	Very Serious	Not serious	Not serous	Very serious	None	78	83	**Not pooled**	**Not pooled**	⨁ Very Low	Critical
Note: Downgraded 2 points for high RoB; downgraded 2 points for imprecision (very small sample size).												
**PICO 1.7 does antihypertensive treatment (I), compared to avoiding this intervention (C ) reduce risk of Mood disorder?**												
1	Randomised trial	Very Serious	Not serious	Not serous	Very serious	None	31	NA	**Not pooled**	**Not pooled**	⨁ Very Low	Critical
Note: Downgraded 2 points for high RoB; downgraded 2 points for imprecision (very small sample size).												

MACE: major adverse cardiac event; RoB: Risk of Bias

The most relevant of these for PICO1 is the INFINITY prospective, randomized, open-label, blinded end points trial.^20^ This study included 199 patients aged ≥75 years (mean age 80.5 years) with systolic hypertension (systolic BP [SBP] of 150 to 170 mm Hg if taking ≥1 antihypertensive drugs or >170 mm Hg on 0 to 1 antihypertensive drug at screening) and MRI evidence of WMH (≥0.5% lesion volume corrected for intracranial cavity volume size, mean WMH volume at baseline 20.6 mL). Patients were randomized to receive intensive treatment targeting a 24-hour mean SBP of ≤130 mm Hg, versus standard treatment targeting ≤145 mm Hg with antihypertensive therapies. Primary outcomes were changes in mobility (gait speed) and accrual of WMH volume after three years. Secondary outcomes included changes in cognitive function and adverse events. The mean 24-hour SBP was 127.7 mm Hg in the intensive treatment group and 144.0 mm Hg in the standard treatment group. Over three years, the risk of stroke, cognitive outcomes, mortality, and changes in gait speed (**PICOs 1.1, 1.2, 1.4, 1.6**), did not differ significantly between treatment groups. Major non-fatal adverse cardiovascular events (**PICO 1.5**) were less common in the intensive vs. standard treatment group (P<0.01).

The study population of the two other trials did not strictly match our definition of ccSVD. In a preliminary open trial,^22^ Pantoni et al. enrolled 31 patients with dementia (DSM-IIIR), leukoaraiosis on the CT scan with a Hachinski ischaemic score >7,^23^ and global deterioration on the Global Deterioration Scale^24^ and the Sandoz Clinical Assessment Geriatric Scale.^25^ These patients were administered for 1 year a 90 mg daily dose of Nimodipine, a calcium channel blocker used primarily for its vasodilating properties on brain vessels, and not as an antihypertensive drug. Primary and secondary outcomes are not clearly distinguished. During follow-up a significant improvement was observed in the Hamilton depression rating scale and the Corsi bock tapping test, while no difference was observed for other cognitive tests or for the Akhtar Disability rating scale score (**PICOs 1.2, 1.3, 1.7**). The randomized, double-blinded and placebo-controlled clinical trial by Zhang et al.^21^ included 732 hypertensive patients (SBP ≥140 mm Hg and/or diastolic BP (DBP) ≥90 mm Hg, or self- reported use of BP-lowering medications in the last 2 weeks), aged ≥60 years and taking hydrochlorothiazide as their baseline medication. These were randomized using a 2 × 2 factorial design with antihypertensive (telmisartan vs. placebo) and lipid-modulating (low-dose rosuvastatin vs. placebo) arms. Participants were not specifically selected for having ccSVD, but all underwent a brain MRI, showing a mean WMH volume at baseline of 5.3 mL (10.4% had a WMH Fazekas score ≥2^1^). Results by WMH severity were not provided. The decline over time in mini mental state examination (MMSE) and Dementia Rating Scale (DRS) scores did not differ significantly between the telmisartan and placebo groups, nor did the incidence of cognitive impairment, which was defined by a MMSE ≤23 at any annual follow-up visit or a decline by ≤3 points between any two annual follow-up visits, and/or a DRS score ≤123 at any annual follow-up visit (**PICO 1.2**).

## Additional information

Given the dearth of direct evidence, we also reviewed indirect evidence for PICO1. Studies evaluating the impact of antihypertensive treatment on some PICO1 outcomes (stroke, MACE, mortality, dementia) outside the context of SVD are numerous, while little to no data is available on the impact of antihypertensive treatment on other PICO1 outcomes (mobility, mood disorders).

Hypertension is the strongest acquired risk factor for stroke (**PICO 1.1**) and, because of its high frequency, about half of all strokes are attributable to hypertension.^26^ Many clinical trials and meta-analyses have long demonstrated that lowering BP reduces the risk of stroke, coronary heart disease events (in primary and in secondary prevention), and the risk of death.^27,28^ The SPRINT trial showed that among 9361 patients aged ≥50 years (mean 67.9), with an SBP between 130 and 180 mm Hg, and at high risk for cardiovascular events but without diabetes or a history of stroke, targeting an SBP <120 mm Hg, as compared with <140 mm Hg, resulted in lower rates of fatal and nonfatal major cardiovascular events and death from any cause, although significantly higher rates of some adverse events were observed in the intensive-treatment group.^29^ Among 2636 SPRINT participants aged ≥75 years, targeting an SBP <120 mm Hg compared with an SBP <140 mm Hg also resulted in significantly lower rates of fatal and nonfatal MACE and death from any cause.^30^ Based on this and other evidence, some but not all clinical guidelines in recent years have recommended BP targets lower than the standard targets of less than 140/90 mm Hg.^31,32^ Indeed, there remains some controversy on the risk/benefit ratio of very low SBP targets.^33^ The earlier ACCORD trial for instance, in 4733 participants with type 2 diabetes at high vascular risk, had not found any significant difference in the primary outcome of nonfatal myocardial infarction, nonfatal stroke, or death from cardiovascular causes in the intensive (SBP <120 mm Hg) vs. the standard (SBP <140 mm Hg) BP lowering group (although the risk of stroke, a secondary outcome, was significantly reduced), while the risk of adverse events was significantly higher.^34^

The association of hypertension and antihypertensive drugs with dementia (**PICO 1.2**) has long been controversial. Early systematic reviews suggested that the association of BP with cognitive decline and dementia is complex and differs according to age and follow-up duration.^35^ Overall, the relationship was stronger in studies with a longer follow-up and with BP measurements in midlife;^36,37^ while studies with a short follow-up or cross-sectional studies in late-life were less consistent, with no association or even hypertension being associated with a lower risk of dementia.^38^ While exposure to hypertension in midlife probably better reflects the total exposure to elevated BP throughout the life course, studies in late-life are also exposed to: (i) reverse causation with BP drops induced by concomitant chronic diseases, including neurodegenerative diseases; (ii) selective survival due to early death from vascular disease of individuals exposed to high BP levels.^39^ The most recent systematic review and meta-analysis of observational data on the association of BP with cognitive impairment and dementia (209 prospective studies), showed that midlife hypertension is associated with 1.19 to 1.55 times increased risk of cognitive disorders, starting at >130 mm Hg SBP on a dose-response analysis.^40^ In late life, high SBP, low DBP, increased BP variability and orthostatic hypotension were all associated with increased dementia risk, although encouragingly, use of antihypertensive drugs was associated with a 21% reduction in dementia risk.^40^ This provides clear evidence that relationships between BP and cognitive disorders are both age- and BP type-dependent, suggesting that caution is still needed until more data are available for older patients.

The most recent and largest Individual Participant Data (IPD) meta-analysis, with the longest follow-up, combined 6 population-based longitudinal cohort studies (n=31090, age >55 years, 7 to 22 years follow-up).^41^ This study found that in participants in the highest BP stratum (≥140/90 mm Hg) using any versus no antihypertensive drug was associated with a lower risk of dementia (HR 0.88, 0.79–0.98, p=0.019) and any dementia, with no difference between drug classes. No significant effect of antihypertensives on dementia was observed in participants in the normal BP stratum (<140/90 mm Hg).^41^ A systematic review of 27 longitudinal studies (21 observational, 6 RCTs, n=56866), found no clear evidence of dementia reduction for any antihypertensive drug versus placebo in participants aged > 65 years, nor any evidence for a drug class being superior to others, except for inconsistent evidence of benefit for diuretics (across follow-up time, comparator group, and outcome).^42^

Randomized trials of antihypertensive drugs that have included an evaluation of dementia or cognitive function (**PICO 1.2**), always as a secondary outcome, also reported contrasted results, usually with a short follow-up period compared with the slow pathophysiological processes leading to dementia.^43^ In the SPRINT-MIND trial (N=9361), although intensive BP lowering (SBP<120 mm Hg vs <140 mm Hg) did not result in a significant reduction in the risk of probable dementia for non-diabetic participants at high vascular risk, a lower risk of the secondary combined outcome of mild cognitive impairment or dementia was observed (median follow-up 5.11 years).^44^ Of note, the ACCORD-MIND trial (N=4733), which used a similar BP target (SBP<120 mm Hg) in diabetic patients, observed a greater decline in total brain volume (p=0.01) but no difference in cognitive decline compared with the standard BP intervention group at 40 and 80 months.^45,46^ In the largest systematic review and meta-analysis combining 14 RCTs (96158 participants),^47^ BP lowering with antihypertensive agents versus controls reduced the risk of dementia or cognitive impairment (12 trials; 92135 participants; mean age 69 years, mean follow-up 4.1 years; odds ratio [OR], 0.93 [95% CI, 0.88–0.98]; absolute risk reduction, 0.39% [0.09%-0.68%]; I^2^ = 0.0%) and slowed down cognitive decline (8 trials; 67476 participants; 20.2% vs 21.1% of participants over a mean trial follow-up of 4.1 years; OR, 0.93 [0.88–0.99]; absolute risk reduction, 0.71% [0.19%-1.2%]; I^2^ = 36.1%).^47^

**Figure 2.**
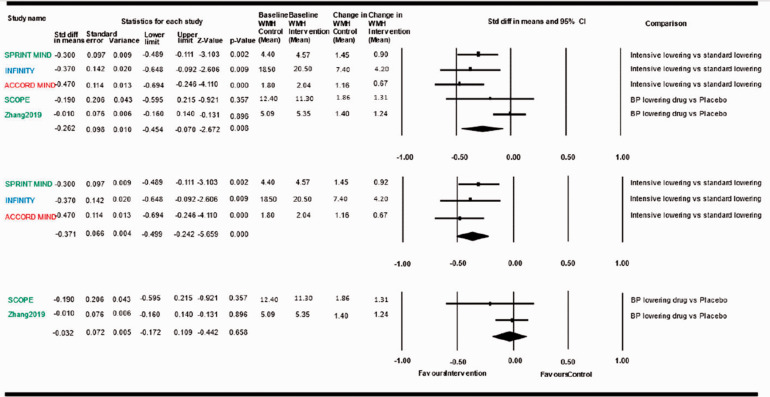
Meta-analysis of effects of BP lowering in RCTs on WMH progression. Blue: ccSVD and hypertensive study population; Red: Diabetic study population; Green: Hypertensive and/or high vascular risk study population; WMH: white matter hyperintensity.

In the absence of robust specific evidence on the clinical impact of antihypertensive treatment in patients with ccSVD, we examined the potential generalizability of the aforementioned observations in broader aging populations to patients with ccSVD. High BP (SBP and DBP), as well as BP variability,^48^ have been consistently and strongly associated with WMH burden and WMH progression, as well as with the presence of lacunes in several population-based studies, with evidence for causality from Mendelian randomization analyses.^49,50^ Moreover, several trials have examined the impact of antihypertensive treatment on WMH progression (either as a primary outcome,^20,21^ or, in most instances, as a secondary outcome in a subset of the trial participants^46,51–55^) and have been combined in two systematic reviews and meta-analyses.^56,57^ The latest, largest systematic review^57^ combined seven trials on 2693 participants, with a mean follow-up duration between 24 and 60 months.^20,21,51–55^ Overall, compared with the control group, patients in the (intensive) BP management group had a slower progression of WMH, with a pooled intergroup standard mean difference (SMD) for WMH change of −0.22 (95% CI: −0.35, −0.09, I^2^ = 63%). For three studies comparing intensive vs. standard BP targets (SPRINT-MIND, ACCORD-MIND, INFINITY),^20,54,55^ the pooled SMD was −0.37 (95% CI:-0.50, -0.24, I^2^ = 0%), while the pooled SMD of studies comparing active antihypertensive medication vs placebo (PROGRESS, PROFESS, SCOPE, Zhang et al)^21,51–53^ was only −0.08 (95% CI: −0.17, 0.01, I^2^ = 0%) and non-significant. Meta-regression analyses showed that the reduction in WMH progression was proportional to the magnitude of intensive BP control (β=−0.028, P <0.001), while the mean age of the study population and the duration of follow-up were not associated with the effect measures. As our guideline focuses on ccSVD, we conducted a new meta-analysis excluding the two trials on stroke patients (PROGRESS and PROFESS),^52,53^ seeking additional data from study authors where necessary (Figure 2). The main characteristics of the five trials included in this meta-analysis are described in Table 3. Overall, compared with the control group, patients in the (intensive) BP management group had a slower progression of WMH, with a pooled intergroup SMD for WMH change of −0.26 (95% CI: −0.45, −0.07, I^2^ = 72%). For the three studies comparing intensive vs. standard BP targets (SPRINT-MIND, ACCORD-MIND, INFINITY),^20,54,55^ the pooled SMD was identical to the last published meta-analysis by Lai et al.,^57^ while the pooled SMD of studies comparing active antihypertensive medication vs placebo (SCOPE, Zhang et al).^21,51^ was only −0.03 (95% CI: −0.17, 0.11, I^2^ = 0%) and non-significant. Table 3 also reports baseline and absolute change values for BP and WMH volume. Meta-regression analyses did not show any significant modifying effect of baseline WMH volume (in all study participants, p=0.84) or of absolute change in WMH volume over follow-up (in controls, p=0.64) on the association between (intensive) BP lowering and WMH progression. Of note, these meta-regressions should be interpreted with caution, given (i) the small number of trials included; (ii) the fact that WMH volume likely has a skewed distribution.

**Table 3.** Characteristics of studies assessing the impact of antihypertensive treatment with WMH progression in PICO 1.

	ACCORD-MIND^55^	SCOPE^51^	SPRINT-MIND^54^	INFINITY^20^	Zhang 2019^21^
Intervention	SBP <120 mmHg vs <140mmHg	Candesartan vs placebo, target BP target BP <160/90 mmHg, changed to <160/90 mmHg in 1999	SBP <120mmHg vs <140mmHg	24-hour SBP ≤130 mmHg vs ≤145 mmHg	Telmisartan 40–80 mg vs Placebo
Study population	Diabetes mellitus, high cardiovascular risk, and an SBP 130–180 mm Hg	70–89 years; BP: 90–99/160–179 mmHg; untreated or thiazide treated	Age ≥ 50 years, no history of diabetes or stroke, increased cardiovascular risk	ccSVD (WMH, ≥0.5% lesion volume), age≥75 years, systolic hypertension	hypertensive, aged ≥60 years and taking hydrochlorothiazide
Follow-up duration, months	40 (SD, NR)	47.3	47.6	36	59.8
N participants (intervention / control group)	314	92 (45 / 47)	670 (355 / 315)	199 (99 / 100)	732 (366 / 366)
Mean age (+/-standard deviation), years	61.7 ± 4.9	77 ± 4	67.3 ± 8.2	80.5	70.7
SBP (+/-standard deviation) at baseline, mmHg (intervention / control group)	138.7 ± 17.5	167 ± 8 / 88 ± 7	136.0 ± 17.0 / 138.2 ± 15.8 ^a^	147.8 ± 13.4 / 150.3 ± 13.4	156.1 ± 9.6 / 156.4 ± 9.8
Change in SBP, mmHg (all or intervention / control group)	−19.7	−26	−15.3 / −3.3	−17.1 / −4.3^b^	−17.3 / −12.4
Method for WMH volume quantification	In house automated software	In house automated software	In house automated software	In house automated software	Freesurfer
WMH vol at baseline, mL (intervention / control group)	2.04 ± 2.85 / 1.80 ± 2.22	11.3 ± 12.4 / 12.4 ± 15.9^c^	4.57 (4.00–5.14) / 4.40 (3.80–5.00)	20.5 ± 17.2 / 18.5 ± 14.8^c^ (ITT)	5.35 / 5.09
Absolute progression of WMH vol, mL (intervention / control group)	0.67 ± 0.95 / 1.16 ± 1.13	1.31 ± 3.24 / 1.86 ± 3.25^c^	0.92 (0.69–1.14) / 1.45 (1.21–1.70)	4.2 ± 5.7 / 7.4 ± 10.1^c^ (ITT)	1.24 / 1.40
Progression of WMH volume percentage	0.073 ± 0.103% / 0.126 ± 0.123%*(% of TBV)*^d^	0.057% (0.39–1.51%) / 0.072% (0.29–1.15%)*(% of TBV)*	0.066% (0.050–0.082%) / 0.104% (0.087–0.122%) *(% of ICV)*^e^	0.29% / 0.48%*(% of ICV)*	0.11% / 0.13% *(% of ICV)*

^a^Values for participants who completed MRI follow-up.

^b^24 hour systolic BP, intention-to-treat; values are mean SD or median (IQR).

^c^Provided by the authors.

^d^Calculated using baseline TBV from Williamson et al.^46^

^e^Calculated using ICV reported in Nasrallah et al.^54^

ITT: Intention to treat. BP: blood pressure; WMH: white matter hyperintensities, ICV: intracranial volume, TBV: total brain volume.

Overall, there is evidence indicating that antihypertensive medication slows the progression of ccSVD, especially intensive BP lowering. Given the strong causal association of WMH burden with stroke and dementia risk,^5,49^ and the high prevalence of WMH in the population with increasing age, one can speculate that the impact of antihypertensive medication on stroke and dementia risk may at least partly be mediated by its impact on ccSVD. The INFINITY trial (N=199), currently the only RCT focusing specifically on ccSVD patients, observed significantly smaller changes in WMH volume over three years in the intensive antihypertensive treatment compared with the standard treatment group (0.29% vs. 0.48%; P=0.03), for a starting volume of 21.1 and 20.2 ml (more WMH than most other studies, Table 3).

Additional studies focussed on ccSVD patients, on larger samples, with a range of ccSVD burden and characteristics, and longer follow-up periods, are warranted to assess more precisely the impact of antihypertensive medication both on ccSVD progression and clinical outcomes. Of note, ongoing trials include: **t**he LEOPOLD trial (NCT02472028), comparing intensive BP lowering (SBP<135 mm Hg) to routine BP management in 820 older ccSVD patients with hypertension and memory complaints (MMSE>20), with WMH progression over 3 years as a primary outcome and secondary clinical endpoints including stroke, cognitive decline, dementia, gait, MACE, and mortality; the CEREBRAL trial^58^ compares the impact of angiotensin-converting enzyme inhibitors and angiotensin II receptor blockers on fatal or non-fatal cerebrovascular events and progression of cerebrovascular lesions in 346 hypertensive patients aged ≥65 years, with any cerebral ischaemic lesion (overt or covert); trials testing the impact of BP lowering drugs on microvascular function and on cerebral autoregulation capacity (NCT03082014; http://www.who.int/trialsearch, NTR6661).

Regarding the following **Evidence-based Recommendation**, please note that according to the GRADE methodology, the QoE of these recommendations is very low for all outcomes since it is based on a small number of small trials with a high risk of bias, imprecision due to very small sample size, as well as indirectness, due either to no control group for comparison or the control group also being given intervention.

However, this is counterbalanced by the strength of the additional information in support of:

- a benefit of antihypertensive treatment in hypertensive patients in the population (many of whom are expected to have ccSVD) to reduce the risk of stroke, cardiovascular events (and possibly, with weaker, recent evidence, also of dementia or cognitive impairment); and
- a benefit of antihypertensive treatment to slow down the progression of ccSVD.

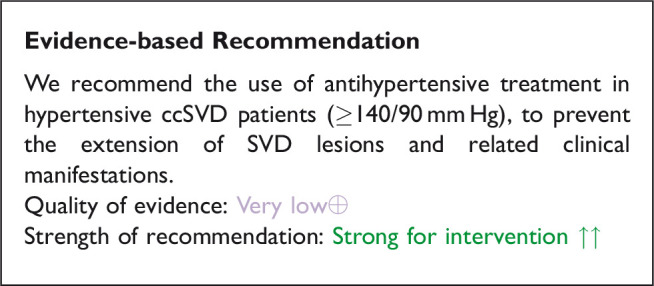


## Rationale for expert consensus statement

Additional studies are warranted in patients with ccSVD to determine the ideal BP level below which extension of SVD lesions and related clinical manifestations are best prevented, and whether specific antihypertensive treatments should be preferred over others, as there is currently no evidence for that. In the meantime, we do not suggest a specific target BP in patients with ccSVD and hypertension, but rather that BP is well controlled according to standard targets set in local practice guidelines. As reduced cerebral blood flow and impaired cerebral autoregulation has been demonstrated in ccSVD patients, there have been some concerns that in patients with extensive ccSVD, very intensive BP lowering could decrease perfusion, thus further increasing ischaemic damage.^59^ The PRESERVE trial in 111 patients with lacunar stroke, confluent WMH and hypertension does not support this hypothesis, as intensive SBP lowering (<125 mm Hg) was not associated with reduced cerebral blood flow (substudy, N=62) or with increase in white matter damage assessed by diffusion tensor imaging MRI.^60–62^ It is also reassuring that in the INFINITY trial the rates of serious falls and syncope were comparable between the treatment groups, suggesting that a target mean 24-hour ambulatory SBP of 130 mm Hg is safe in older individuals with hypertension. However, additional studies are needed on larger groups of ccSVD patients, with clinical endpoints including vascular events, cognition, mobility and mood, and taking into account the effect of antihypertensive medication on the different BP traits (SBP, DBP, BP variability, orthostatic hypotension). Outside the specific context of ccSVD, evidence from a recent Cochrane systematic review, which included six RCTs (9484 participants), did not support lower BP targets (<135/85 mm Hg) as compared to standard BP targets (<140-160/90-100 mm Hg) in people with hypertension and established cardiovascular disease(myocardial infarction, stroke).^31^ This systematic review suggested that, in addition to a lack of benefit for the lower BP target in terms of total or cardiovascular mortality, total cardiovascular events, and serious adverse events, a numerical increase in total mortality prompted caution. In the SPS3 trial, which focused on 3020 patients with overt SVD (recent lacunar stroke), although the use of an SBP target <130 mm Hg, compared to a target of 130-149 mm Hg did not lead to a significant reduction in all stroke, it was associated with a significantly reduced rate of intracerebral haemorrhage (p=0.03), and treatment-related serious adverse events were infrequent.^33^

Another unsolved question to explore in the future is whether reducing BP ought to be recommended in all patients with ccSVD, regardless of whether they have clinically defined hypertension (>140/90 mm Hg), as is a current practice in secondary stroke prevention since the PROGRESS trial. In older populations a non-negligible proportion of ccSVD is not caused by hypertension, often related to cerebral amyloid angiopathy, although both mechanisms may co-exist and interact.^63^ Recently a two-sample Mendelian randomization analysis suggested a causal association of increasing BP with higher WMH volume even among persons without clinically defined hypertension.^49^

Expert Consensus Statement

- All group members suggest that: BP should be appropriately monitored and well controlled. Provided that BP is well controlled we cannot advise any specific antihypertensive treatment.
- Most group members suggest that: For ccSVD patients, there is currently insufficient evidence to systematically advocate targeting BP levels lower than standard targets, although more intensive BP lowering than conventional BP lowering guidelines is associated with slower progression of WMH burden.
- All group members suggest that: In ccSVD patients in whom more intensive BP lowering targets are recommended for other reasons there is no strong evidence to suggest that this could be harmful.
- On current evidence the guideline group unanimously does not support systematic BP lowering in normotensive ccSVD patients.

**PICO 2:** In patients with covert cerebral small vessel disease [WMH and/or lacunes], does antiplatelet treatment, compared to less intense or avoiding this intervention, reduce ischaemic or haemorrhagic strokes (2.1), cognitive decline or dementia (2.2), dependency (2.3), death (2.4), MACE (2.5), mobility (2.6), or mood disorders (2.7)?

## Analysis of current evidence

**Figure 3.**
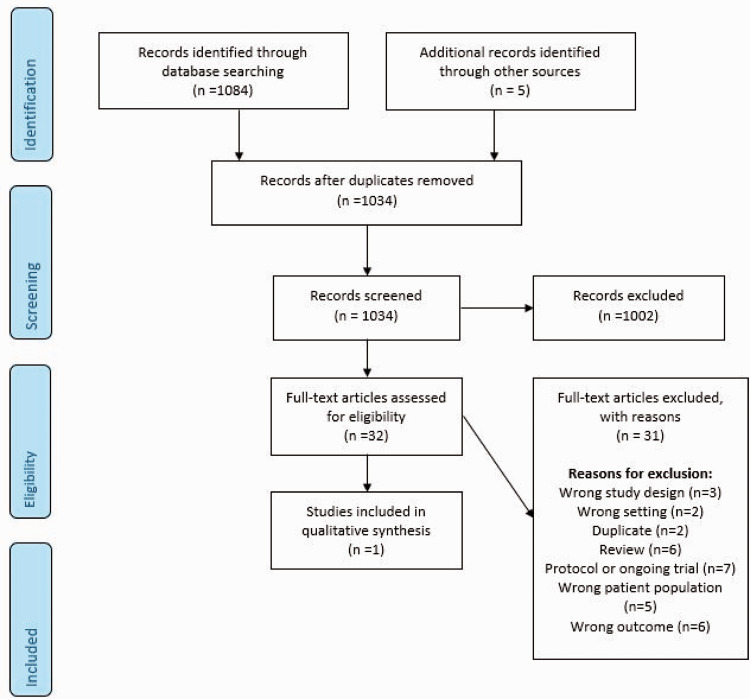
Search results PICO 2

We identified one study in patients with ccSVD (Figure 3, Table 4),^64^ and a second study in a population that can be considered at high risk for ccSVD, where antiplatelet therapy was trialled for preventing the specified clinical outcomes defined in this guideline document.^64,65^ Pooling was not possible, therefore we describe each study and the PICO outcome that is addressed.

**Table 4.** Summary of clinical trial findings relevant for PICO 2.

Outcome	Study	Study design	Population	Age	Intervention	Comparison	Duration of follow-up	IPD Intervention group		IPD control group		OR/HR/RR/or summary of findings	RoB rating
								No. with event	Total in group	No. with event	Total in group		
Stroke	Maestrini 2018	RCT/observational	≥45 years old, presented with at least 1 SBI on MRI	67 (Mean)	ASA 100 mg	Placebo or no therapy	4 years	1	36	2	47	OR: 0.643 (0.056-7.381)^a^	High
Cognitive Decline or Dementia	Maestrini 2018	RCT/observational	≥45 years old, presented with at least 1 SBI on MRI	67 (Mean)	ASA 100 mg	Placebo or no therapy	4 years	Not given	36	Not given	47	No significant difference between intervention and control groups for any cognitive domains investigated.	High
Death	Maestrini 2018	RCT/observational	≥45 years old, presented with at least 1 SBI on MRI	67 (Mean)	ASA 100 mg	Placebo or no therapy	4 years	0	36	1	47	OR: 0.425 (0.017-10.733)	High
MACE	Maestrini 2018	RCT/observational	≥45 years old, presented with at least 1 SBI on MRI	67 (Mean)	ASA 100 mg	Placebo or no therapy	4 years	2	36	1	47	OR: 2.706 (0.236-31.077)	High
Mood disorder	Maestrini 2018	RCT/observational	≥45 years old, presented with at least 1 SBI on MRI	67 (Mean)	ASA 100 mg	Placebo or no therapy	4 years	Not given	36	Not given	47	No significant difference between intervention and control groups.	High

RoB: risk of bias; RCT: randomised controlled trial; IPD: individual participant data; ASA: acetylsalicylic acid (Aspirin); SBI: silent brain infarcts; MRI: magnetic resonance imaging; OR: odds ratio; numbers in parentheses = 95% confidence limits; HR: hazard ratio; RR: risk ratio; MACE: major adverse cardiac event; RoB: risk of bias.^a^Calculated based on IPD data, unadjusted OR.

### Aspirin vs placebo

One randomised, double blind placebo-controlled trial included patients ≥45 years with at least one silent brain infarct (SBI) but no previous clinical cerebrovascular events for randomization to aspirin 100 mg (n=36) or placebo (n=47).^64^ The primary endpoint was the combined endpoint of ischaemic stroke, TIA, and new silent brain infarcts (SBIs) detected at MRI which had occurred in nine controls (19.1%) and two subjects (5.6%) in the ASA group (p=0.10) after four years. A new stroke was observed in 2/47 (4.3%) subjects in the control group and 1/36 (2.8%) in the ASA group (OR: 0.64 (0.06–7.38), calculated based on individual patient data in the publication) (**PICO 2.1**). There were 1/47 (2.1%) deaths in the control group and 0/36 (0%) deaths in the aspirin-treated group (OR: 0.43 (0.02–10.73)); **PICO 2.4**). There were 1/47 (2.1%) myocardial infarctions in the control group and 2/36 (5.6%) in the aspirin-treated group (OR: 2.71 (0.24–31.08)); **PICO 2.5**). Findings should be interpreted with care as the number of incident ischaemic strokes and myocardial infarctions were small with wide confidence intervals including one. In addition, there were important differences in prevalence of hypertension between controls compared to aspirin users (61.7% vs 47.2%, p=0.188). The authors reported no significant differences in secondary endpoints.^64^

### Cilostazol vs no antithrombotic treatment

Cilostazol has weak antiplatelet effects, in addition to other actions. One trial randomized patients with Type II diabetes (which can be considered a population at high risk for SVD) to treatment with cilostazol 100-200 mg per day, n=43 (34 with no brain lesions on baseline MRI) or no antithrombotic treatment, n=46, (27 with no brain lesions on baseline MRI).^65^ After a study period of 3.2 years, symptomatic brain infarcts occurred in two subjects in the control group whereas no stroke occurred in the cilostazol treatment group (**PICO 2.1**). An increased number of infarct-like lesions as detected by MRI at the end of the study was seen in 16 subjects in the control group and two in the cilostazol group.^65^

The two identified studies^64,65^ are both small and even though a tendency to better clinical outcomes was seen in both studies, they provide insufficient support for a general recommendation of antiplatelet treatment in subjects with ccSVD to avoid new clinical events (Table 5**)**, which only includes the study by Maestrini et al^64^ because we did not consider the study of patient with diabetes mellitus by Shinoda-Tagawa et al^65^ to be a “true” primary prevention study.

**Table 5.** Quality of results in trials relevant for PICO 2.

Certainty assessment							№ of patients		Effect		Certainty	Importance
№ of studie**s**	Study design	Risk of bias	Inconsistency	Indirectness	Imprecision	Other considerations	Antiplatelet Therapy	No antiplatelet therapy	Relative(95% CI)	Absolute(95% CI)		
**PICO 2.1 does antiplatelet treatment (I), compared to avoiding this intervention (C ) reduce risk of ischaemic or haemorrhagic strokes?**												
1	Randomised trial	Very serious	Not serious	Not serious	Very serious	None	36	47	**Not pooled**	**Not pooled**	⨁ Very Low	Critical
Note: Downgraded 2 points for high Risk of Bias; downgraded 2 points for imprecision (very small sample size).												
**PICO 2.2 does antiplatelet treatment (I), compared to avoiding this intervention (C ) reduce risk of cognitive decline or dementia?**												
1	Randomised trial	Very serious	Not serious	Not serious	Very serious	None	36	47	**Not pooled**	**Not pooled**	⨁ Very Low	Critical
Note: Downgraded 2 points for high Risk of Bias; downgraded 2 points for imprecision (very small sample size).												
**PICO 2.4 does antiplatelet treatment (I), compared to avoiding this intervention (C ) reduce risk of death?**												
1	Randomised trial	Very Serious	Not serious	Not serous	Very serious	None	36	47	**Not pooled**	**Not pooled**	⨁ Very Low	Critical
Note: Downgraded 2 points for high Risk of Bias; downgraded 2 points for imprecision (very small sample size).												
**PICO 2.5 does antiplatelet treatment (I), compared to avoiding this intervention (C ) reduce risk of MACE?**												
1	Randomised trial	Very Serious	Not serious	Not serous	Very serious	None	36	47	**Not pooled**	**Not pooled**	⨁ Very Low	Critical
Note: Downgraded 2 points for high Risk of Bias; downgraded 2 points for imprecision (very small sample size).												
**PICO 2.7 does antiplatelet treatment (I), compared to avoiding this intervention (C ) reduce risk of Mood disorder?**												
1	Randomised trial	Very Serious	Not serious	Not serous	Very serious	None	36	47	**Not pooled**	**Not pooled**	⨁ Very Low	Critical
Note: Downgraded 2 points for high Risk of Bias; downgraded 2 points for imprecision (very small sample size).												

MACE: major adverse cardiac event.

We found no randomized trials of antiplatelet agents in patients with ccSVD that provided evidence of effects on the outcomes of cognitive decline or dementia (**PICO 2.2**), dependency (**PICO 2.3**), mobility (**PICO 2.6**) or mood disorders (**PICO 2.7**).

## Additional information

There are several stroke secondary prevention studies examining if antiplatelet treatment is beneficial for preventing progress of ccSVD. Some of these studies focused mostly on neuroradiological features but there are also studies that examined clinical outcomes. There are also primary prevention studies examining non-clinical outcomes of SVD, mainly progress of SVD on MRI.

### Secondary prevention after lacunar stroke

#### Antiplatelet agents vs placebo and dual antiplatelet agents vs aspirin or placebo

The SPS3 trial of dual vs single antiplatelet drugs given for a mean of 3.4 years after clinical lacunar stroke found that dual versus single antiplatelet drugs increased death, and did not prevent stroke^66^ or cognitive decline.^67^

A systematic review of antiplatelet drugs in secondary prevention after lacunar stroke (including SPS3) included 17 trials (42234 participants, mean age 64.4 years, 65% male) and follow up from 4 weeks to 3.5 years.^68^ Compared with placebo, any single antiplatelet agent reduced any stroke (risk ratio [RR] 0.77, 0.62–0.97, 2 studies) and ischaemic stroke (RR 0.48, 0.30–0.78, 2 studies), but not the composite of any stroke, myocardial infarction, or death (RR 0.89, 0.75–1.05, 2 studies). Other antiplatelet agents (ticlodipine, cilostazol, and dipyridamole) compared with aspirin, gave no consistent reduction in stroke recurrence (RR 0.91, 0.75–1.10, 3 studies) and dual versus single antiplatelet therapy did not confer clear benefit (any stroke RR 0.83, 0.68–1.00, 3 studies; ischaemic stroke RR 0.80, 0.62–1.02, 3 studies; composite outcome RR 0.90, 0.80–1.02, 3 studies).^68^

An observational study compared a PGE1 inhibitor (alprostadil injections for 10 days followed by beraprost tablets up to 3 months) + aspirin with aspirin alone given for one year in patients with prior lacunar ischaemic stroke and found a reduction in ischaemic stroke (RR 0.74, 95%CI 0.65–0.88) without any increase in haemorrhagic stroke (RR 0.92, 95%CI 0.79–1.13). However, the observational nature of the study and limited reporting of methods and results reduce the reliability of these data.^69^

#### Cilostazol vs other antiplatelet agents or placebo

The a subgroup analysis of the ECLIPse trial found no effect on WMH change after 90 days of cilostazol (a weak antiplatelet agent) treatment in lacunar stroke patients.^70^

The LACI-1 trial is a small (n=57) trial that found that cilostazol administered over 11 weeks to patients with lacunar stroke was associated with less progression of WMH compared to patients not treated with cilostazol.^71^

### Secondary prevention after ischaemic stroke of different types

#### Dual antiplatelet therapy

A substudy in the CHANCE trial of dual vs single antiplatelet therapy given for three weeks for secondary stroke prevention in 787 patients with minor stroke with MRI at baseline found no interaction between WMH and antiplatelet therapy and recurrent stroke or functional outcome at 3 months.^72^ Although the main CHANCE trial found short term dual antiplatelet therapy to be beneficial in preventing recurrent ischaemic stroke after TIA or any subtype of minor ischaemic stroke, this substudy in patients with MRI did not show specific benefit from dual vs single antiplatelet agents in patients with high versus low burden of SVD.

#### Cilostazol in ischaemic stroke

A systematic review of cilostazol in secondary stroke prevention suggested a reduced hazard (including less systemic bleeding) and better stroke prevention vs placebo or other single antiplatelet agents, including a suggestion of more benefit in lacunar stroke.^73^

The PICASSO study compared cilostazol to aspirin in ischaemic stroke patients with a previous intracerebral haemorrhage or multiple microbleeds. There was no difference in white matter lesion volume change between 254 subjects in the aspirin group and 251 in the cilostazol group.^74^ However, cilostazol reduced the incidence of any stroke in patients with mild-to-moderate white matter changes but not in patients with severe white matter changes, suggesting that cilostazol might be effective in the earlier stages of SVD.^75^

### Primary prevention

The ASPREE trial tested the effect of 100 mg aspirin versus placebo on vascular events in 19114 healthy people aged 70 years or older (or ≥65 years of age among blacks and Hispanics in the United States) over 4.7 years of follow-up, finding no effect on reducing cardiovascular disease (HR 0.95, 95%CI 0.83–1.08), ischaemic stroke (HR 0.89, 95%CI 0.71–1.11),^76^ dementia (HR 0.98, 95%CI 0.83–1.15), disability (HR 0.85, 95%CI 0.70–1.03), or the combined endpoint of death, dementia, or physical disability (HR 1.01, 95% CI 0.92–1.11).^77^ However, aspirin did increase major haemorrhage (HR 1.38, 95%CI 1.18–1.62), intracranial haemorrhage (HR 1.5, 95% CI 1.11–2.02),^76^ and deaths from all causes, mainly cancer (HR 1.14, 95% CI 1.01–1.29).^78^ This implies that giving aspirin to older people, many of whom are likely to have SVD lesions, and who do not have a history of stroke or cardiovascular disease is unlikely to provide benefit and may cause harm.

The Women’s Health Initiative Memory Study of Magnetic Resonance Imaging Study is an observational prospective study of long-term aspirin use after enrolment of patients into a RCT of hormone replacement therapy.^79^ In can be debated whether this was a true primary prevention study because the aspirin users more often had cardiovascular disease. After eight years, MRI scans showed no significant difference in WMH volume between chronic aspirin users and nonusers in unadjusted or adjusted analyses, even though chronic aspirin users had nonsignificantly (4.8%, 95% CI:−6.8–17.9%) larger WMH volumes after covariate adjustment.^79^

Several ongoing studies are noted below for a more comprehensive overview of ongoing treatment studies of cerebral SVD.

A study in South Korea compares aspirin vs cilostazol in 254 patients with moderate or severe cerebral white matter changes and 1 or more lacunar infarction(s) on MRI, NCT 01932203 with a primary outcome of change in WMH volume between baseline and 104 weeks on MRI With secondary clinical outcomes (ischaemic strokes, all vascular events, cognition, and disability).^80^

An ASPREE substudy (ENVIS-ion) described in 2012, will examine cognitive function and brain MRI at baseline and after 3 years of treatment in 600 subjects aged 70 years or older randomized to aspirin or placebo, NCT01038583.^81^ Another ASPREE substudy, the Study of Neuro-cognitive Outcomes, Radiological and Retinal Effects of Aspirin in Sleep Apnoea (SNORE-ASA), assesses cognition, brain MRI and retinal changes in 296 subjects at study entry and after 3 years.^82^

The LACI-2 study is a prospective randomised open label blinded endpoint (PROBE) trial of 400 patients with lacunar ischaemic stroke testing the effect of cilostazol (and in partial factorial design, isosorbide mononitrate), on clinical (vascular events, cognition, dependency, death) and imaging markers of SVD at one year.^83^ The COMCID trial in Japan tests effect of cilostazol in patients with MCI^84^ and the Cilostazol in Retarding Progression of Cerebral White Matter Hyperintensities trial in China is testing the effect of cilostazol WMH volume change with secondary cognitive outcomes (http://www.chictr.org.cn/com/25/hvshowproject.aspx?id=3079).


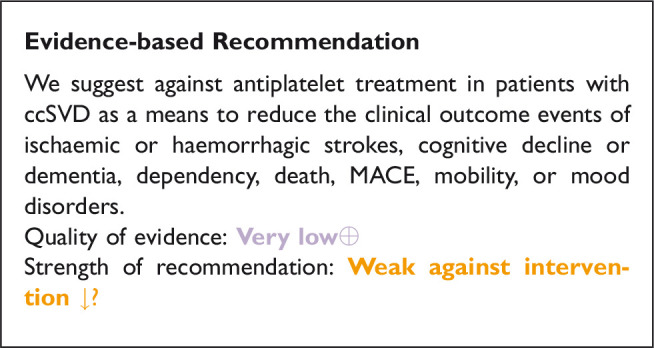


## Rationale for expert consensus statement

Guidelines recommend the use of antithrombotic drugs to reduce the risk of recurrent stroke in patients with TIA or minor stroke, and of recurrent MI, MACE and vascular death in patients with prior overt vascular events.^85,86^ However, we found no evidence to support the use of antiplatelet drugs such as aspirin in patients with ccSVD who did not have any other indication, and some evidence that giving antiplatelet drugs such as aspirin to ccSVD would be hazardous, particularly in older people,^78^ if no other indication for this treatment exists.

There is a need for additional trials to better understand if antiplatelet treatment may have some benefit in ccSVD. However, such trials should only be performed after careful considerations accounting for the results of previous trials. Even though aspirin could be harmful in this situation in older people, other weak antiplatelet drugs which have additional pharmacological effects could be beneficial, such as cilostazol. Furthermore, better recognition and definition of the atypical neuropsychiatric and cognitive symptoms that are associated with ccSVD^10^ and other markers currently in investigation may help identify a group of patients for ‘secondary prevention’, with a high burden of disease and higher risk for future ischaemic events and/or a lower risk of bleeding complications, who might benefit from aspirin or equivalent drugs, and could be tested in clinical trials.

Our literature search shows that the hitherto reported results from RCTs, although usually emanating from small sample sizes, do not support that antiplatelet agents have a beneficial effect on ccSVD and adds weight to the evidence that the main underlying pathophysiology of ccSVD may not be atherothromboembolic in origin but due to other vascular dysfunction mechanisms.^87^

Expert Consensus Statement

Most group members agreed that:

• We advise against use of antiplatelet drugs to prevent clinical outcomes in subjects with ccSVD when no other indication for this treatment exists.

• With current available knowledge, the use of antiplatelet drugs to prevent progression of cerebral SVD may be harmful in older patients (from around ≥70 years of age) if no other indication for this treatment exists.

**PICO 3:** In patients with covert small vessel diseases [WMH and/or lacunes], does lipid lowering treatment, compared to less intense or avoiding this intervention, reduce ischaemic or haemorrhagic strokes (3.1), cognitive decline or dementia (3.2), dependency (3.3), death (3.4), MACE (3.5), mobility (3.6), or mood disorders (3.7).

## Analysis of current evidence

**Figure 4.**
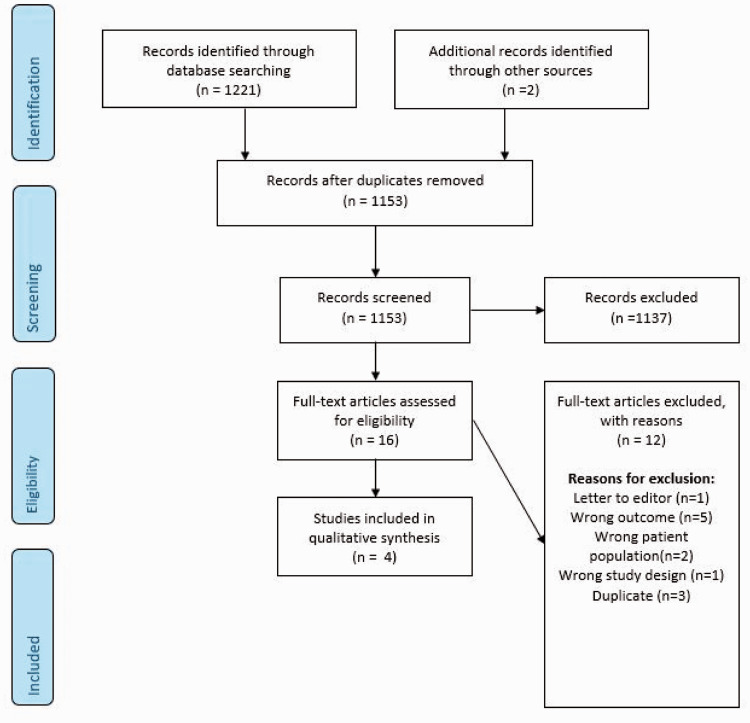
Search results PICO 3.

Three RCTs and one relevant observational study were identified in the literature search of some relevance to ccSVD (Figure 4, Tables 6 and 7). Due to heterogeneity of outcomes, the data were not suitable for pooling in a meta-analysis, so the results are summarized narratively below.

**Table 6.** Summary of clinical trial findings relevant for PICO 3.

Outcome	Study	Study design	Population	Mean Age	Intervention	Comparison	Duration of follow-up	IPD Intervention group		IPD control group		OR/HR/RR/ Summary of findings	RoB rating
								No. with event	Total in group	No. with event	Total in group		
Stroke	Mok 2009	RCT	36- to 75-year-olds with risk factors for Atherosclerosis and mild to moderately elevated fasting LDLcholesterol (LDL-C) of 3.0–5.0 mmol/L.	63 (Median)	Simvastatin 20 mg daily	Placebo	2 years	3	106	3	102	OR: 0.961 (95%CI: 0.189 to 4.876)^a^	Some concerns
	Guo 2020	RCT	Aged ≥75, HTN	78 (Mean)	Rosuvastatin 10mg daily	Placebo	Median of 63 months	Not given	124	Not given	127	HR: 0.560, (95% CI: 0.374 to 0.838)	High
	Guo 2020	Observational	Aged ≥75, HTN	78 (Mean)	Statin	Non-statin	7 Years	Not given	129	Not given	698	HR: 0.598 (95% CI: 0.368 to 0.973)	Moderate
Cognitive Decline or Dementia	Zhang 2019a	RCT	Aged ≥60, HTN	70 (Mean)	Rosuvastatin 10mg daily	Placebo	Median of 63 months	39	366	68	366	OR: 0.513 (95%CI: 0.334 to 0.790)	High
	Zhang 2019b	RCT	Patients with mild cognitive impairment and ccSVD	65 (Mean)	Rosuvastatin+nimodipine	Nimodipine only	6 months	NA	44	NA	44	“The MoCA scores were higher after treatment for both groups, while the Intervention group had a more obvious effect (P<0.05)”	High
Dependency	Zhang 2019b	RCT	Patients with mild cognitive impairment and ccSVD	65 (Mean)	Rosuvastatin+nimodipine	Nimodipine only	6 months	NA	44	NA	44	“The ADL scores were higher after treatment for both groups, while the observation group had a more obvious effect (P<0.05)”	High
MACE	Mok 2009	RCT	36 to 75 years old with risk factors for Atherosclerosis and mild to moderately elevated fasting LDLcholesterol (LDL-C) of 3.0–5.0 mmol/L.	63 (Median)	Simvastatin 20 mg daily	Placebo	2 years	0	106	2	102	OR: 0.189 (95%CI: 0.009 to 3.979)^a^	Some concerns

RoB: risk of bias; RCT: randomised controlled trial; IPD: individual participant data; HTN: hypertension; OR: odds ratio; HR: hazard ratio; RR: risk ratio.

^a^Calculated based on IPD data, unadjusted OR.

**Table 7.** Quality of results in trials relevant for PICO 3.

Certainty assessment							№ of patients		Effect		Certainty	Importance
№ of studies	Study design	Risk of bias	Inconsistency	Indirectness	Imprecision	Other considerations	Lipid lowering treatment	No lipid lowering treatment	Relative (95% CI)	Absolute (95% CI)		
**PICO 3.1 does lipid lowering treatment (I), compared to avoiding this intervention (C ) reduce risk of ischaemic or haemorrhagic strokes?**												
1	Observational	Serious	Very serious	Not serous	Very serious	Plausible confounding would reduce the demonstrated effect	129	698	Not pooled	Not pooled	⨁ Very Low	Critical
2	Randomised trials	Very serious	Not serious	Not serious	Very serious	None	230	229	Not pooled	Not pooled	⨁ Very Low	Critical
Note: Observational: Deducted 2 points for observational evidence; deducted 1 points for risk of bias in study. Deducted 2 points for imprecision (single study, limited sample size) Upgraded 1 point due to plausible confounding reducing demonstrated effect. Randomised Trial: Deducted 2 points for high risk of bias in one study; deducted 2 points for imprecision (small overall sample size).												
**PICO 3.2 does lipid lowering treatment (I), compared to avoiding this intervention (C ) reduce risk of cognitive decline or dementia?**												
2	Randomised trials	Very serious	Not serious	Not serious	Very serious	None	410	410	**Not pooled**	**Not pooled**	⨁ Very Low	Critical
Note: Deducted 2 points for high risk of bias in both studies; deducted 2 points for imprecision (small overall sample size).												
**PICO 3.3 does lipid lowering treatment (I), compared to avoiding this intervention (C ) reduce risk of dependency?**												
1	Randomised trial	Very serious	Not serious	Not serious	Very serious	None	44	44	**Not pooled**	**Not pooled**	⨁ Very Low	Critical
Note: Deducted 2 points for high risk of bias; deducted 2 points for imprecision (small overall sample size).												
**PICO 3.5 does lipid lowering treatment (I), compared to avoiding this intervention (C ) reduce risk of MACE?**												
1	Randomised trial	Serious	Not serious	Not serious	Very serious	None	106	102	**Not pooled**	**Not pooled**	⨁ Very Low	Critical
Note: Deducted 1 point for uncertain Risk of bias; deducted 2 points for imprecision (small overall sample size and wide Confidence Intervals).												

MACE: major adverse cardiac event.

**Table 8.** Summary of findings in trials assessing the effect of lipid lowering on WMH.

	Ji, 2018^91^ Zhang 2019^21^ Guo^90^	PROSPER^93^	ROCAS^165^
Intervention	rosuvostatin 10mg/day	pravastatin	simvastatin 20mg/day
Study population	Chinese, >60years, hypertensive, ie primary	pre-existing coronary, cerebral, peripheral vas dis or risk of vasc dis (smoke, HT, DM); ie primary and secondary	Chinese asymptomatic MCA stenosis, ie primary
Follow-up duration, months	median 61.8	33	24
N participants (intervention / control group) [2]	[2]732(366/366) started - [1]668 (342/326 provided data)	535	208 (106 / 102)
Mean age, years	[2] 70.90 ± 6.28 ; 70.47 ± 6.14	75 =/-3yr	63
lipid at baseline, mmol/l(intervention / control group)	[2] TC 5.13 ± 0.64/ 5.06 ± 0.66; LDL 3.28 ± 0.67 /3.21 ± 0.68 mmol/L	NA	TC 5.9(0.6) v 5.9(0.6); LDL 3.9(0.5) v 3.9(0.5)
Change in lipidmmol/l (intervention / control group)	on graphs in [2]	NA	TC -1.4 v 0, LDL -1.4 v -0.1
Method for WMH volume quantification	volume; Fazekas score	volume	vol
WMH severity at baseline	NA	NA	none (68.8%), mild (18.3%), severe (13%)
WMH vol at baseline, mL (intervention / control group)	5.08 (3.82, 6.77); 5.52 (3.84, 6.68)	1.6 (0.5-5.9) v 1.8 (0.5-5.5)cm3	1.6 (4.6) v 1.1 (3.2) cm3
WMH vol baseline % ICV (Interv/control)	0.42 (0.31,0.55); 0.44 (0.31, 0.54)	NA	NA
Absolute progression of WMH vol, mL (intervention / control group)	1.20+/-0.46 vs 1.72+/-0.43 mL, adjusted P < 0.001	1.0 (0.1-2.9) v 0.9 (0.1-2.9)cm3; MD 1.1 vs 1.1 cm^3,^ P=0.73	0.0 (0.3) v cm3 0.0 (0.0)^a^
Progression of WMH volume percentage	NA	NA	14.0% (30.4%) v 16.8% (47.1%)^b^
Fazekas score change in active vs control group	HR 0.50 (0.34–0.74)	NA	NA
incident Faz >/=2	39 (12.1%) vs. 71 (22.8%), P < 0.001	NA	NA
incident lacunes in control v active group	HR 1.860, 95% CI 1.124e3.078; P ¼.016,	NA	NA
microbleeds	no effect	NA	NA

^a^All patients; for severe WMH group.

^b^The % increase is of the baseline WMH.WMH: white matter hyperintensities; HT: hypertension; DM: diabetes mellitus; MCA: Middle Cerebral Artery; TC: total cholesterol; LDL: low density lipoprotein cholesterol; HR: hazard ratio; CI: Confidence Interval; MD: median.

### Does lipid lowering treatment (I), compared to less intense or avoiding this intervention (C) reduce risk of ischaemic or haemorrhagic strokes?

One study^88^ performed a post-hoc analysis of data from the ROCAS (Regression of Cerebral Artery Stenosis) study,^89^ which was a randomized, double-blind, placebo-controlled trial evaluating the effects of statins upon asymptomatic middle cerebral artery (MCA) stenosis progression among stroke-free individuals. The primary focus of this post-hoc analysis of 207 subjects was to assess WMH progression as an outcome (see below), but the study also reported stroke as an outcome with no difference between groups (3 events in each arm).

Guo et al^90^ reported a combination of a prospective population-based cohort study (N=827) and a subset of patients from a randomized, double-blind, placebo-controlled clinical trial^21,91^ which had a 2x2 factorial design investigating BP lowering and lipid lowering with rosuvastatin 10mg mixed regimen in elderly Chinese individuals (N=227). The primary outcome for this study was WMH progression (see below), but incident stroke was a secondary outcome. The risk of incident stroke was significantly lower in the rosuvastatin group than the placebo group (HR: 0.56, 95% CI: 0.37 to 0.84, P<0.001), a finding replicated in the observational cohort (HR: 0.60, 95% CI: 0.37 to 0.98).

### Does lipid lowering treatment (I), compared to avoiding this intervention (C) reduce risk of cognitive decline or dementia?

We identified two RCTs. The first was a randomized placebo controlled factorial 2x2 design of BP lowering (telmisartan) and lipid lowering (rosuvastatin) in 732 elderly hypertensive Chinese patients who were already taking hydrochlorothiazide.^21^ The MMSE and DRS were secondary outcomes and they both declined more slowly in patients taking rosuvastatin. Of note, the patients in this report are from the same RCT as reported by Ji, 2018^91^ and Guo et al. ^90^

Zhang et al.^92^ reported a RCT of rosuvatatin plus nimodipine versus a control arm of nimodipine alone administered for six months in 120 patients with mild ccSVD. The third outcome listed was MoCA at six months. Data given in Figure 3 (not in text) of the publication suggests the average MOCA score was 18 in the active and 17 in the control group prior to the trial treatment, and were 22 in the treatment group and 19 in the control group after six months’ treatment (P<0.05).

### Does lipid lowering treatment (I), compared to avoiding this intervention (C) reduce risk of dependency?

Zhang et al^92^ also report on Activities of Daily Living. Data from Figure 4 (not given in text) shows ADL scores were higher in both groups at the end of six months treatment than pre-treatment and were highest in the active treatment group (ADL score of 69 in active vs. 61 in control groups, P<0.05)

### Does lipid lowering treatment (I), compared to avoiding this intervention (C) reduce death?

We found no RCT or observational studies related to this question.

### Does lipid lowering treatment (I), compared to avoiding this intervention (C) reduce MACE?

One aforementioned study^88^ performed a post-hoc subgroup analysis of a secondary outcome in patients with asymptomatic MCA stenosis and found no difference in the rate of myocardial infarction between the intervention (simvastatin) and the control arm.

### Does lipid lowering treatment (I), compared to avoiding this intervention (C) reduce risk of mobility impairment?

We found no RCT or observational studies related to this question.

### Does lipid lowering treatment (I), compared to avoiding this intervention (C) reduce risk of mood disorders?

We found no RCT or observational studies related to this question.

## Additional information

We identified six studies (of which three publications report on patients from the same trial^21,90,91^) that assessed the effect of lipid lowering on SVD lesions progression on neuroimaging, in addition to the two publications mentioned above. We believe it relevant to report the results of these trials, although the clinical implications of reducing the burden of ccSVD on neuroimaging are not yet established (Table 8).

A brain MRI and cognitive function substudy of a RCT enrolled 732/1244 Chinese hypertensive patients aged >60 years randomized to rosuvastatin 10mg/day or placebo^91^ from which clinical outcomes were provided in Zhang et al.^21^ Of 688 patients with pre- and post-treatment period MRI and included in the analysis, WMH volume and the Fazekas rating scale were determined at baseline and after a median follow-up of 61.8 months on brain MRI. The outcomes included changes in WMH volume, new-incident Fazekas scale score >2, new-incident lacunes, and new-incident microbleeds. The RCT found a lower increase in WMH volume in the rosuvastatin vs control groups, respectively 1.20+/-0.46 vs 1.72+/-0.43 mL, adjusted P < 0.001. Compared with the rosuvastatin group, the risk of new-incident Fazekas scale score >2 was higher (HR 2.15, 95% CI 1.44, 3.20; P < 0.001), and of new-incident lacunes was higher (HR 1.86, 95% CI 1.12, 3.08; P =0.016) in the placebo group. There was no significant effect of rosuvastatin on the risk of new-incident microbleeds.

A substudy of the PROSPER trial in 535 participants tested the effect of pravastatin vs placebo on WMH progression over 33 months, finding no effect (MD 1.1 vs 1.1 cm^3^, P=0.73).^93^

Mok et al, in the ROCAS RCT trial substudy,^88^ found no effect of simvastatin 20mg daily versus placebo for two years on WMH progression in 208 patients with asymptomatic MCA stenosis and a median age 63 years, but most patients (about 70%) had very few or no WMH at baseline. In the few patients with a high WMH burden (2-3 cm^3^ at baseline), there was some evidence of reduced WMH progression in the patients allocated to simvastatin (n=12, 23.2% WMH increase) versus control (n=15, WMH increase 58.2%), adjusted P=0.019.

Guo et al^90^ examined WMH progression in a subset of patients from the same clinical trial of statins vs control reported above.^21,91^ They included 227 subjects from the clinical trial and 781 subjects from a cohort study split according to statin (about one fifth) use or not (about 4/5ths) users, with average follow-up of 63 months. In the 227 participants in the RCT, half allocated to rosuvastatin 10mg daily and half to placebo, the risks of progression of WMH (HR: 0.41, 95% CI: 0.23 to 0.72, P<0.001) and lacunes (HR: 0.42, 95% CI: 0.26 to 0.68, P<0.001) were significantly lower in the rosuvastatin group than the placebo group after adjustment for confounders BP and incident stroke. A similar pattern of reduction in WMH and lacune progression was seen in the observational study amongst statin users vs non-users.

The Mayo Clinic Study of Aging (MCSA)^94^ is a prospective observational population-based study which included 1160 residents in Olmsted County, Minnesota (USA) aged >65 years. The study included neuropsychological assessment, cerebral PET, blood and cerebrospinal fluid biomarkers. Subjects were classified as statin-treated, statin-untreated (≤3 months of treatment), and long-term statin-treated (≥5 years of treatment). The study assessed WMH on structural brain MRI and found that statin use did not influence neuroimaging markers, with no difference between lipophilic and hydrophilic statins. The study provided no information on clinical outcomes.

A prospective case-control study performed in a memory clinic included 474 patients classified as with “no cognitive impairment”, “cognitive impairment no dementia”, and “dementia”.^95^ Patients underwent MRI scans assessing lacunes, microbleeds, and WMH after a 2-year follow-up. The study found that use of statins was associated with a decreased risk of incident lacunes (OR: 0.15, 95% CI: 0.04, 0.61) but not with WMH progression (OR: 1.09, 95%CI 0.53–2.28) or with new microbleeds (OR: 1.81, 0.73–1.99). This study provided no clinical outcomes.

**Figure 5.**
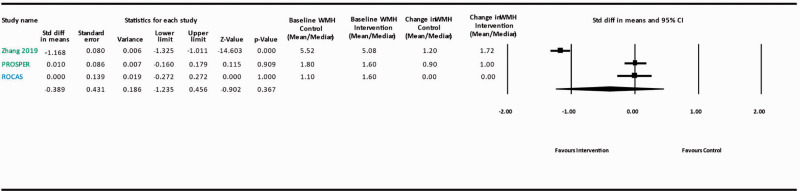
Meta-analysis Lipid lowering and WMH progression. Blue: Covert Small Vessel Disease study population; Green: Hypertensive and/or high vascular risk study population.

We conducted a random effects meta-analysis of WMH progression in the three trials comparing 'absolute progression of WMH volume' for each group (control vs intervention). We used WMH volume mean and SD, converting medians to means where needed (Figure 5).^96^ The standardised mean difference was –0.40, 95%CI -1.24, 0.46, p=0.37, with substantial between trial heterogeneity (I-^2^= 98.294, Q=117.204). The sparse information from the trials and skewed distribution of WMH volumes reduces the reliability of this analysis.

Overall, the six papers (four studies) suggest a possible role of statins, in particular rosuvastatin, in preventing the progression of neuroimaging signs of ccSVD, but provide no clinical outcomes and three of the four studies are further limited by their observational design.

The ACCORD MIND trial^46^ randomised 1538 participants with type 2 diabetes (who could be considered a high risk group for ccSVD) to lipid lowering with a fibrate versus placebo. There was no difference in Digit Symbol Substitution Test mean scores between fibrate versus placebo lipid groups (lipid difference between means, −0.08 [−0.92 to 0.76]; P=0.85) or for the three other cognitive tests, at 40 months. Among 503 participants with baseline and follow-up MRI, there was no difference in total brain volume at 40 months between fibrate (change from baseline -12.9, -16.1 to -9.8) and control (change from baseline -14.1, 95%CI -17.1, -11.1) adjusted P=0.59.

A systematic review and meta-analysis of four trials testing the effect of intensive versus guideline lipid lowering on post-stroke cognitive impairment, comprising 623 participants with stroke including lacunar stroke found those allocated to intensive statin versus control had better cognitive outcomes (mean difference on ACE-R score, 1.34, 95%CI 0.15–2.53, p=0.03).^97^


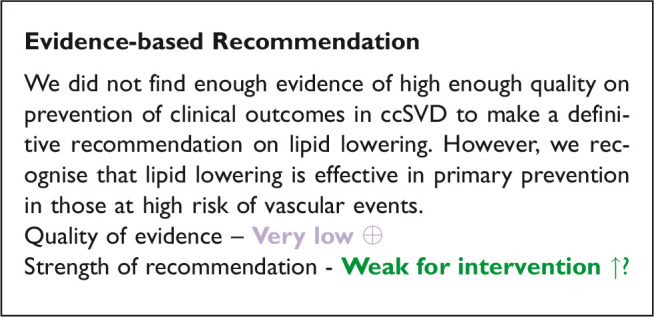


## Rationale for expert consensus statement

Primary prevention of cardiovascular disease is recommended in subjects at the highest cardiovascular risk, including at older ages, to prevent vascular events.^98,99^ Lipid lowering by several agents in older subjects reduced stroke, MI, and cardiovascular death with similar effect to those aged <75yrs; however, limited data suggested no effect on cognitive outcomes.^100^ Although the role of elevated plasma lipid levels might be less relevant in ccSVD in comparison with, for example, atherothrombotic strokes, it is important to assess whether lipid lowering treatments can decrease the risk of cardiovascular outcomes in patients with ccSVD.

Expert consensus statement

The group members were narrowly in favor that:

• Lipid lowering with statins could be considered in patients with ccSVD, even when no other indication for statin treatment exists, with the aim of delaying the progression of ccSVD, although the clinical implications of this delayed progression remain to be proven.

**PICO 4:** In patients with covert cerebral small vessel diseases [WMH and/or lacunes], do lifestyle interventions [smoking cessation, weight reduction, dietary interventions, physical exercise, cognitive/social interventions, sleep/CPAP, or a mixture of these], compared to less intense or avoiding these interventions, reduce ischaemic or haemorrhagic strokes (4.1), cognitive decline or dementia (4.2), dependency (4.3), death (4.4), MACE (4.5), mobility (4.6), or mood disorders (4.7).

## Analysis of current evidence

**Figure 6.**
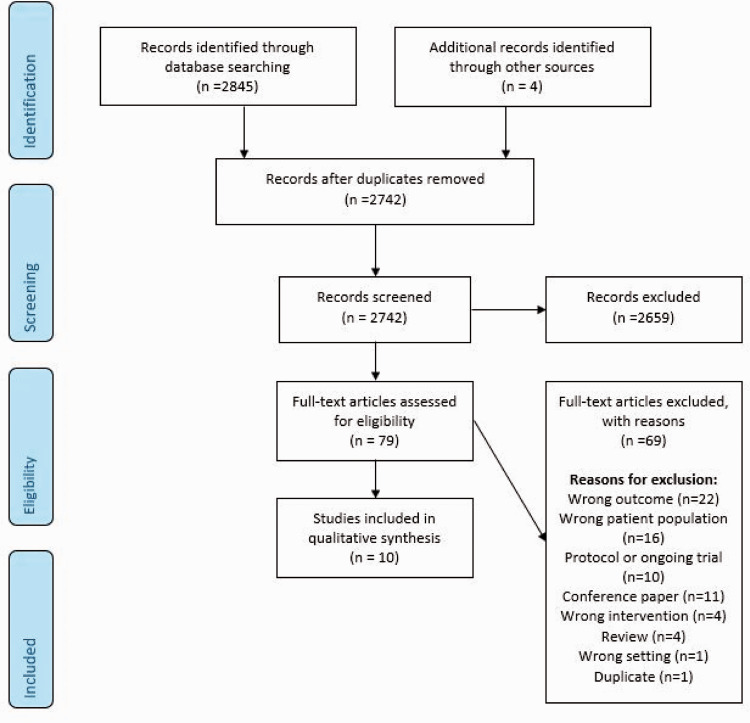
Summary of search results PICO 4.

Lifestyle interventions aim to control obesity, hypertension, mental health, diabetes, dyslipidaemia and sleep obstructive disorders by avoiding harmful habits, increasing aerobic physical activities, adopting dietary measures, or improving cognitive skills. Addressing lifestyle factors are strongly recommended (Level I recommendation) in the primary prevention of stroke and cardiovascular diseases.^101,102^ The World Health Organization Guidelines recommend physical activity and smoking cessation for the primary prevention of cognitive decline and dementia, but the level of evidence is moderate for the efficacy of dietary intervention and low for cognitive and social interventions.^103^ Therefore, we have assessed whether evidence exists about lifestyle interventions in the population with ccSVD, who might benefit more from lifestyle interventions given the tight association between SVD and vascular risk factors, but also might be prone to higher risk of adverse events e.g. from falls due to physical activity compared to healthy subjects. It should be noted that all RCTs identified were graded as having a serious or very serious risk of bias and low certainty surrounding the estimate of efficacy **(**Figure 6, Tables 9 and 10). We included in our literature assessment RCTs and prospective observational studies since cross-sectional studies would not allow assessing the effect of interventions, but only associations.

**Table 9.** Summary of clinical trial findings relevant for PICO 4.

Outcome	Study	Study design	Population	Mean Age	Intervention	Comparison	Duration of follow-up	IPD Intervention group		IPD control group		OR/HR/RR/Beta/Summary of findings	RoB rating
								No. with event	Total in group	No. with event	Total in group		
Stroke	Landman 2021	Observational	Aged 50-85 with established ccSVD	66	Physically active	Physically inactive	9 years	26	251	48	252	HR: 0.58, 95%CI: 0.36 to 0.96	Moderate
Cognitive Decline or Dementia	Tang 2019	RCT	People with subcortical VCIND	64	Computerized cognitive training	Active control group	6 months	NA	30	NA	30	Significant difference between groups observed for performance on MoCA at 7 weeks (Mean difference: 3.441, p-value: 0.013) and for performance on the Boston Naming Test (mean diff=3.187, p=0.028) favouring intervention group. No significant differences were observed between group performance on any cognitive tests at 6 months.	High
	Bowman 2020	RCT	Age 75 and older with MRI derived total WMH ≥ 5 cm3	81	Marine n-3 polyunsaturated fatty acids (n-3 PUFA)	Soybean oil placebo	3 years	NA	Individual group numbers not given. Total sample size=72	NA	Individual group numbers not given. Total sample size=72	Three-year changes in executive cognitive function (z-scores) were not different between intervention and control groups (p =0.24).	High
	Wong 2020	RCT	Age ≥65 years, presence of ≥2 lacunar infarcts and/or a rating of ≥2 on the ARWMC scale	Not given	Aerobic Dance	Simple stretching and health education on cognition, mood, physical, and daily functioning	36 weeks	NA	54	NA	56	Performance on test of executive functioning significantly favoured the intervention group over the control group at 24 weeks; however, this performance difference was non-significant at 36 weeks. Performance improvements were also observed for ‘Delayed recognition’ performance at 24 weeks which remained significant at 36 weeks. Delayed recall performance was significantly greater in the intervention group by comparison to the control group at 36 weeks.	High
	Liu-Ambrose 2016 (PROMOTE trial)	RCT	Adults with a clinical diagnosis of mild SIVCI	74	Aerobic exercise	Usual care plus education on cognitive and everyday function	12 months	NA	35	NA	35	Performance on ADAS-Cog was significantly better for the treatment group as compared to the control group at end of intervention (Adjusted between group difference: -1.71 95%CI=-3.15 to -0.26). However, this performance difference was no longer significant 12 months after the intervention began and there were no significant differences in cognitive performance between intervention group and controls on any other cognitive measures.	Some concerns
	Hsu 2015 (PROMOTE trial)	RCT	Adults with a clinical diagnosis of mild SIVCI	72	Aerobic exercise	Usual care plus education on cognitive and everyday function	6 months	NA	10	NA	11	Significantly better performance observed on the ‘incongruent condition’ of the Flanker task favouring the intervention group upon completion of the intervention (Mean difference: 0.063, p=0.007).	High
	Wang 2020	RCT	Aged 40 to 70, presence of traditional vascular risk factors and cognitive impairment, diagnosis of persistent insomnia	63	Improved sleep (via Trazodone)	Placebo	4 weeks	NA	16	NA	14	Trazodone treatment resulted in greater improvement in the concentration and recall abilities than the placebo (P < 0.05).	High
	Gons 2011	Observational	Aged between 50 and 85 years; and ccSVD on neuroimaging	66	Duration of smoking cessation	Never smokers (reference group)	Cross-sectional	NA	Former smokers: 275	NA	Never smokers: 149	Significant trend (p=0.04) identified towards better cognitive performance in which longer duration of smoking cessation was positively related to higher mean cognitive z scores.	Serious
	Frederiksen 2014 (LADIS study)	Observational	age 64–85 years, presence of ARWMC	73	Physically active	Physically inactive	3 years	NA	213	NA	69	Significant association for superior executive functioning subgroup scores favouring those who were physically active (Beta=0.11, 95%CI= 0 to 0.22)	Critical
	Verdelho 2012 (LADIS study)	Observational	Age 64–85 years, presence of ARWMC	74	Physically active	Physically inactive	3 years	NA	409	NA	230	HR: 0.71 (95% CI, 0.524–0.949)	Critical
	Landman 2021	Observational	Aged 50-85 with established ccSVD	66	Physically active	Physically inactive	9 years	NA	251	NA	252	No significant relationship between physical activity and executive functioning (-0.039, 95%CI: -0.157–0.079, P=0.52)	Serious
Dependency	Wong 2020	RCT	Age ≥65 years, presence of ≥2 lacunar infarcts and/or a rating of ≥2 on the ARWMC scale	Not given	Aerobic Dance	Simple stretching and health education on cognition, mood, physical, and daily functioning	36 weeks	NA	54	NA	56	No significant difference between intervention and control groups observed.	Some concerns
	Liu-Ambrose 2016	RCT	Adults with a clinical diagnosis of mild SIVCI	74	Aerobic exercise	Usual care plus education on cognitive and everyday function	12 months	NA	35	NA	35	No significant difference between intervention and control group and measure of activities of daily living (Adjusted between-group difference=1.34, 95%CI=-0.68 to 3.37)	Some concerns
Death	Landman 2021	Observational	Aged 50-85 with established ccSVD	66	Physically active	Physically inactive	9 years	NA	251	NA	252	HR: 0.69, 95%CI:0.49–0.98	Moderate
Mobility	Wong 2020	RCT	Age ≥65 years, presence of ≥2 lacunar infarcts and/or a rating of ≥2 on the ARWMC scale	Not given	Aerobic Dance	Simple stretching and health education on cognition, mood, physical, and daily functioning	36 weeks	NA	54	NA	56	Significant difference favouring the intervention group observed at week 12, on the timed-up-and-go test. However, there was no significant treatment effect on physical functioning at weeks 24 and 36.	Some concerns
Mood Disorder	Wong 2020	RCT	Age ≥65 years, presence of ≥2 lacunar infarcts and/or a rating of ≥2 on the ARWMC scale	Not given	Aerobic Dance	Simple stretching and health education on cognition, mood, physical, and daily functioning	36 weeks	NA	54	NA	56	No significant difference observed between intervention and control groups.	High

RoB: risk of bias; RCT: randomised controlled trial; IPD: individual participant data; OR: odds ratio; HR: hazard ratio; RR: risk ratio; WMH: white matter hyperintensities; ccSVD: covert cerebral small vessel disease; VCIND: vascular cognitive impairment no dementia; ccSVD: cerebral small vessel disease; ARWMC: age-related white matter changes; SIVCI: subcortical ischaemic vascular cognitive impairment; MoCA: Montreal Cognitive Assessment.

**Table 10.** Quality of results in trials relevant for PICO 4.

Certainty assessment							№ of patients		Effect		Certainty	Importance
№ of studies	Study design	Risk of bias	Inconsistency	Indirectness	Imprecision	Other considerations	Life-style interventions	No life-style interventions	Relative (95% CI)	Absolute (95% CI)		
**PICO 4.1 does physical activity(I), compared to avoiding this intervention (C ) reduce risk of ischaemic or haemorrhagic stroke?**												
1	Observational	Not serious	Not serious	Not serious	Very serious	None	251	252	Not pooled	Not pooled	⨁ Very Low	Critical
Note: Downgraded 2 points due to observational evidence; downgraded 2 points due to imprecision (1 study, limited sample size)												
**PICO 4.2.1 does physical activity (I), compared to avoiding this intervention (C ) reduce risk of cognitive decline or dementia?**												
2	Observational	Very serious	Not serious	Not serious	Serious	None	660	482	Not pooled	Not pooled	⨁ Very Low	Critical
2	Randomised trials	Very serious	Not serious	Not serious	Very serious	None	89	91	Not pooled	Not pooled	⨁ Very Low	Critical
Notes: Observational: Downgraded 2 points due to observational evidence; Downgraded 2 points for high risk of bias in studies; downgraded 1 point for relatively limited sample size. Randomised Trial: Downgraded 2 points for high risk of bias in studies; downgraded 2 points for imprecision (small samples; mainly pilot studies).												
**PICO 4.2.2 does smoking cessation (I) compared to avoiding this intervention (C ) reduce risk of cognitive decline or dementia?**												
1	Observational	Very serious	Not serious	Not serious	Very serious	None	275	149	**Not pooled**	**Not pooled**	⨁ Very Low	Critical
Notes: Downgraded 2 points due to observational evidence; downgraded 2 point for risk of bias; downgraded 2 points for imprecision (small sample size).												
**PICO 4.2.3 does better sleep (I) compared to avoiding this intervention (C ) reduce risk of cognitive decline or dementia?**												
1	Randomised trial	Very serious	Not serious	Very serious	Very serious	None	16	14	**Not pooled**	**Not pooled**	⨁ Very Low	Critical
Note: Downgraded 2 points due to high risk of bias; Downgraded 2 points for indirectness (study is a drug to improve sleep in insomniacs); downgraded 2 points for imprecision.												
**PICO 4.2.4 do diet supplements (I) compared to avoiding this intervention (C) reduce risk of cognitive decline or dementia?**												
	Randomised trial	Very serious	Not serious	Not serious	Very serious	None	36	36	**Not pooled**	**Not pooled**	⨁ Very Low	Critical
Note: Downgraded 2 points due to high risk of bias; downgraded 2 points due to imprecision.												
**PICO 4.2.5 does cognitive training (I) compared to avoiding this intervention (C ) reduce risk of cognitive decline or dementia?**												
1	Randomised trial	Very serious	Not serious	Not serious	Very serious	None	30	30	**Not pooled**	**Not pooled**	⨁ Very Low	Critical
Note: Downgraded 2 points due to high risk of bias; downgraded 2 points due to imprecision.												
**PICO 4.3 does physical activity (I), compared to avoiding this intervention (C ) reduce risk dependency**												
2	Randomised trials	Serious	Not serious	Not serious	Very serious	None	89	91	**Not pooled**	**Not pooled**	⨁ Very Low	Critical
Note: Downgraded 1 points due to unclear risk of bias; downgraded 2 points due to imprecision.												
**PICO 4.4 does physical activity (I), compared to avoiding this intervention (C ) reduce risk of death?**												
1	Observational	Serious	Not serious	Not serious	Very serious	None	251	252	**Not pooled**	**Not pooled**	⨁ Very Low	Critical
Note: Downgraded 2 points due to observational evidence; ddowngraded 1 points due to moderate risk of bias; downgraded 2 points due to imprecision.												
**PICO 4.6 does physical activity (I), compared to avoiding this intervention (C ) reduce risk of Mobility impairment?**												
1	Randomised trial	Serious	Not serious	Not serious	Very serious	None	54	56	**Not pooled**	**Not pooled**	⨁ Very Low	Critical
Note: Downgraded 1 points due to unclear risk of bias; downgraded 2 points due to imprecision.												
**PICO 4.7 does physical activity (I), compared to avoiding this intervention (C ) reduce risk of mood disorder?**												
1	Randomised trial	Very serious	Not serious	Not serious	Very serious	None	54	56	**Not pooled**	**Not pooled**	⨁ Very Low	Critical
Note: Downgraded 2 points due to unclear risk of bias; downgraded 2 points due to imprecision.												

### Physical exercise

Two RCTs and three relevant observational studies of physical exercise were identified in the literature search. Due to varying outcomes, the data were not suitable for pooling in meta-analysis, so the results are summarized narratively below.

#### Physical exercise and ischaemic or haemorrhagic strokes

The literature search did not find any RCTs investigating the impact of physical exercise on the incidence of ischaemic or haemorrhagic strokes in individuals with ccSVD.

An observational analysis from the Radboud University Nijmegen Diffusion tensor and Magnetic resonance imaging Cohort (RUNDMC) study with a total of 503 participants with SVD on neuroimaging without dementia (some participants may have had TIA or minor stroke) suggested that higher baseline physical activity as assessed with a questionnaire is related to lower incidence of cerebrovascular events (composite endpoint consisting of TIAs, ischaemic and haemorrhagic strokes and vascular dementia) (adjusted HR: 0.58, 95%CI: 0.36–0.96) over 9-year follow-up. However, there was no association between physical activity and lacunar stroke or WMH progression.^104^

#### Physical exercise and cognitive decline or dementia

Two RCTs provided results of the influence of physical interventions on cognitive decline.

A single-blinded RCT (Promotion of the Mind Through Exercise, PROMOTE) compared a 6-month, thrice-weekly, progressive aerobic exercise training with usual care and education in 71 individuals with SVD (35 per group). At the end of the treatment period, the intervention group had significantly improved global cognitive performance (ADAS-Cog) compared to the control group (-1.71 point difference, 95%CI: -3.15–-0.26), but the difference was not significant after additional 6-month follow-up. The intervention had no significant effect on executive functions (EXIT-25).^105^ A subgroup analysis from the same RCT (10 and 11 per group) showed that subjects randomised to aerobic training significantly improved in flanker task performance, compared to controls, suggesting improved selective attention and response inhibition.^106^

Another RCT compared the efficacy of a 24-week aerobic dance training relative to stretching and health education in 110 older adults (54 and 56 per group) with SVD on multiple cognitive tests administered at 12, 24 and 36 weeks. Significant effects of exercise were reported on executive function (trails test) at 24 weeks, and on delayed recognition (list learning test) at 24 and 36 weeks. Group differences were non-significant in other tests measuring global cognition, memory or processing speed.^107^

An observational 3-year follow-up study, the Leukoaraiosis and Disability Study (LADIS), included 639 individuals with age-related WMH and no disability at enrolment. Self-reported physical activity at baseline was associated with reduced risk of cognitive decline and incident dementia (HR 0.64; 95% CI, 0.48–0.85), and vascular dementia in particular (HR 0.42; 95% CI, 0.22–0.80), independently of confounders including WMH severity and previous and incident stroke.^108^ Domain-specific cognitive functions were analysed in a subgroup of 282 subjects who had not progressed to MCI or dementia. Physical activity was associated with higher baseline scores of processing speed and executive functions, and with less decline in executive functions at follow-up.^109^

#### Physical exercise and dependency

Dependency in activities of daily living was addressed in two RCTs,^105,107^ neither of which found a significant effect of physical exercise on functional abilities.

#### Physical exercise and death

No RCTs addressing the effect of physical exercise on mortality were identified. The observational RUNDMC study has reported that in 503 participants with SVD, higher baseline physical activity level as evaluated with a questionnaire was related to a lower all-cause mortality (adjusted HR: 0.69, 95%CI: 0.49–0.98) over 9-year follow-up.^104^

#### Physical exercise and MACE

The literature search did not identify any RCTs or observational studies addressing this question.

#### Physical exercise and mobility

The RCT comparing the effect of 24-week aerobic dance training with stretching and education (54 and 56 per group) reported a significant treatment effect on mobility as evaluated with the timed-up-and-go test at 12 weeks, but not in walking capacity or balance, and none of the group differences were longer significant at 24 and 36 weeks.^107^

#### Physical exercise and mood disorders

The aforementioned RCT comparing the effect of 24-week aerobic dance training with stretching and education (54 and 56 per group) found no treatment effect on mood as evaluated with the 15-item Geriatric Depression Scale.^107^

### Other lifestyle interventions (non-physical activity)

#### Non physical activity lifestyle interventions and risk of haemorrhagic or ischaemic stroke

The literature search did not identify any RCTs or high quality observational studies addressing this question.

#### Non physical activity lifestyle interventions and risk of cognitive decline or dementia

**Cognitive training** – A RCT (the Cog-vaccine trial^110^) randomized 60 patients with subcortical vascular impairment but no dementia to compare the intervention of a 7 week program of multidomain adaptive computerized cognitive training with an active control of a programme of fixed attention and processing speed tasks. At the end of the 7 week period, those in the intervention arm showed a significant improvement in the MoCA test, but this difference was not maintained at 6 month follow up.

**Dietary supplements –** Bowman et al^111^ performed a randomised double blind placebo controlled trial (total n=102) of fish oil (marine n-3 polyunsaturated fatty acids) compared with a placebo of soybean oil in older patients with evidence of WMH accumulation. Executive cognitive function, measured as an exploratory secondary outcome did not differ between the groups after three years.^111^

**Sleep interventions** – we found one study^112^ that performed a randomised double blind placebo controlled trial of trazodone for four weeks in patients with vascular risk factors and evidence of SVD on brain scanning who had insomnia. Forty patients were recruited, 30 completed the trial (mean age 65 years) and the authors reported no significant change in total MoCA (the primary outcome at four weeks) but did report small significant improvements in the concentration and recall items of the MoCA for the intervention group (see Table 9).

#### Non physical activity lifestyle interventions and risk of dependency

The literature search did not identify any RCTs or high quality observational studies addressing this question.

#### Non physical activity lifestyle interventions and risk of death

The literature search did not identify any RCTs or high quality observational studies addressing this question.

#### Non physical activity lifestyle interventions and risk of MACE

The literature search did not identify any RCTs or high quality observational studies addressing this question.

#### Non physical activity lifestyle interventions and risk of mobility

The literature search did not identify any RCTs or high quality observational studies addressing this question.

#### Non physical activity lifestyle interventions and risk of mood disorder

The literature search did not identify any RCTs or high quality observational studies addressing this question.

## Additional information

Direct evidence of the impact of life-style interventions on clinical outcomes in ccSVD is sparse. RCTs with participants with memory complaints or MCI and no definition of SVD at enrolment (NeuroExercice, AIBL Active) have found no intervention effects of aerobic exercise on cognitive or imaging outcomes.^113,114^ However, an RCT with elderly individuals at-risk for dementia from the general population (FINGER) has shown that a multidomain intervention including diet, exercise, cognitive training and vascular risk monitoring has beneficial effects in improving and maintaining cognitive functioning.^115^ A pooled analysis of 322 individuals from two RCTs targeting lifestyle and vascular risk factors with multi-domain interventions to prevent post-stroke cognitive impairment found limited evidence of benefit on attention but not on other cognitive domains.^116^ An RCT (AFIVASC) is ongoing to assess the efficacy of a 6-month physical activity program on neuropsychological and neuroimaging outcomes in patients with vascular cognitive impairment.^117^

Among community-dwelling older individuals, the observational population-based Northern Manhattan Study has suggested that moderate or heavy physical activity at baseline is associated with lower prevalence of subclinical brain infarcts but not WMH on MRI scan 6 years later.^118^ Another prospective observational study, The Shiga Epidemiological Study of Subclinical Atherosclerosis, in healthy Japanese men has reported a relationship between higher average step count at baseline and lower burden of WMH and lacunar infarcts, but not microbleeds on MRI scans approximately 6 years later.^119^

Existing guidelines^101^ promote lifestyle factors such as physical activity (moderate to vigorous intensity aerobic physical activity at least 40 min/day 3 to 4 days/week is recommended), diet rich in fruit and vegetables, Mediterranean diet supplemented with nuts, overweight reduction and quitting smoking (assisted by counselling in combination with drug therapy) to reduce the risk of stroke in adults with no history of stroke or TIA.^101^ The beneficial effects of physical exercise are clear in the general population and current evidence does not suggest any harm of such activity in ccSVD.

The role of dietary intervention in the prevention of stroke was summarised in a recent review.^120^ The review found that B-group vitamins (folic acid, B6, B12), niacin, or vitamin D supplementations had no benefit on recurrent stroke; folic acid alone or with low dose vitamin B12, but not with high doses of B12, can reduce stroke risk in geographical areas where dietary folate is low. Omega-3 fatty acids overall had no effect on stroke incidence or for ischaemic stroke and increased the risk for haemorrhagic stroke. Mediterranean diet decreased the risk of stroke and the DASH diet effectively reduced BP. The review concluded that patients at risk of stroke should be advised to follow a Mediterranean-style diet and to increase fruit and vegetable consumption. Homocysteine lowering with vitamins B6, B9, or B12 reduced the risk of stroke, but not myocardial infarction, death, or serious adverse events compared with placebo.^121^ Another review found no effect of vitamin C for primary prevention of cardiovascular disease.^122^ A further review found no effect of omega-6 fats for the primary and secondary prevention of cardiovascular disease except for MI.^123^

A cross-sectional study^124^ suggested that smoking cessation is associated with improved white matter structural integrity and better cognition compared with not stopping smoking.


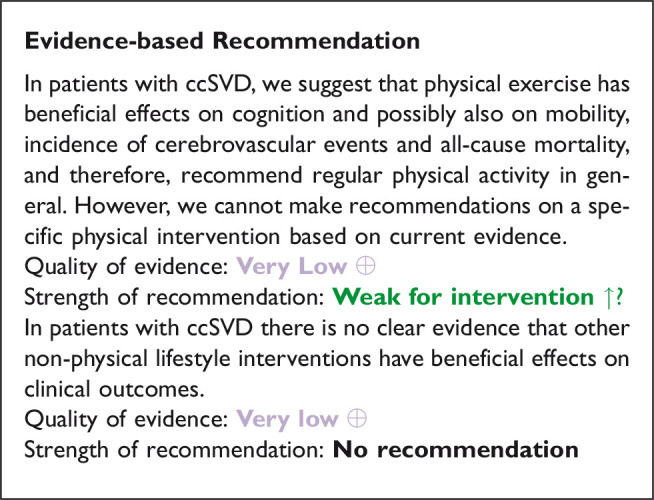

Expert consensus statement

All group members suggest that:

• There is no direct evidence to suggest that any specific lifestyle interventions prevent clinical outcomes in patients with ccSVD.

• However it is reasonable to promote healthy lifestyle interventions as recommended in primary prevention for vascular disease (including but not limited to maintaining healthy body weight, promoting exercise, avoiding smoking and excess alcohol, eating a healthy, balanced diet) in patients with ccSVD.

**PICO 5:** In patients with covert cerebral small vessel diseases [WMH and/or lacunes] who may (or may not) require glucose lowering therapies, do drugs which reduce plasma glucose levels, compared to less intense or avoiding these interventions, reduce ischaemic or haemorrhagic strokes (5.1), cognitive decline or dementia (5.2), dependency (5.3), death (5.4), MACE (5.5), mobility (5.6), or mood disorders (5.7)?

## Analysis of current evidence

**Figure 7.**
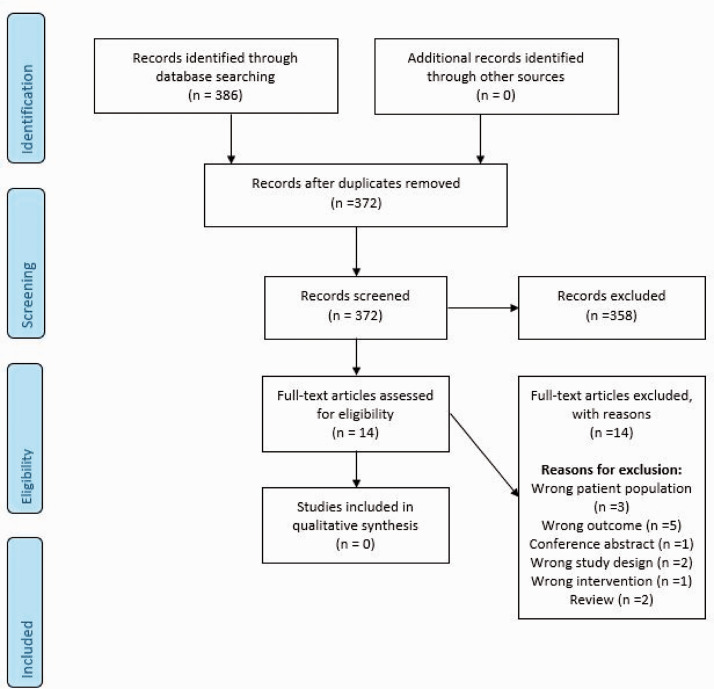
Summary of search results PICO 5.

The literature search did not identify any RCT or observational study directly addressing these PICO questions. No study investigated specifically glucose lowering therapies in patients with ccSVD (Figure 7). We therefore examined whether there was any evidence to guide ccSVD management in people living with diabetes, although relevant studies addressing this topic also proved to be scarce.

In a Cochrane review of 32 studies, four RCTs comparing metformin alone to the combination of metformin with second or third generation sulphonylurea showed that non-fatal stroke occurred in 14/2098 (0.7%) participants under metformin versus 8/2995 (0.3%) under combined treatments (RR 2.21, 95% CI 0.74 to 6.58; P = 0.15).^125^ No benefit was detected, either, for global mortality, cardiovascular mortality or fatal stroke and there were no data on dementia.^125^

In the ACCORD-MIND substudy cohort of 502 people with diabetes followed over 40 months, WMH progression did not differ between intensive treatment with target HbA1C <6% and standard treatment with target HbA1C between 7 and 8% (0.92 ± 1.31 vs 0.93 ± 1.06 cm3, p = 0.917).^55^ An earlier analysis found more WMH in the intensive glycaemic control group at 40 months (difference 0.18cm^3^ - 95% CI 0.16–0.2, p=0.015), particularly in patients aged less than 60 years.^126^ However, there was less loss of total brain volume at 40 months in the intensive treatment than in the control group (difference in volume 4.6, 95% CI 2.0 to 7.3, cm^3^ respectively, p=0.0007).^126^ The protective effect of intensive glycaemic control on preserving brain volume was confirmed in later analyses, where however an interaction between BP lowering and glycaemic control on total brain volume was detected.^46^ However, there was no difference between intensive and guideline glycaemic control groups in any of four cognitive tests (e.g. Digit Symbol Substitution Test score difference in mean scores at 40 months 0.32, 95% CI –0.28 to 0.91; p=0.2997) in the 2957/2977 MIND participants with cognitive data, and the overall MIND trial had stopped early due to excess mortality in the intensive glycaemic control arm.^126^

## Additional information

Diabetes has been repeatedly but inconstantly associated with the extent of WMH or with their progression in several population-based studies.^127^ Moreover, markers of diabetic retinal or kidney microangiopathy as well as diabetic neuropathy have been also associated with a larger extent of WMH.^127^ In the Maastricht study, a population-based cohort of 2228 participants, both HbA1c, fasting and 2-h post-load plasma glucose levels were related to the volume of WMH and presence of lacunes in individuals with pre- or type 2 diabetes.^128^ Similarly, type 2 diabetes was associated with more lacunes, WMH and brain volume loss in 851 patients presenting to a memory clinic with vascular cognitive impairment.^129^ Among 1713 participants of the Atherosclerosis Risk in Communities - Neurocognitive Study (ARIC-NCS), only patients with diabetes with HbA1c ≥7.0% but not diabetic or non-diabetic individuals with HbA1c <7.0% had an increased burden of WMH.^130^ Among 618 non-demented elderly Medicare recipients from Northern Manhattan, HbA1c as a continuous or categorical variable was also found to be associated with a higher number of brain infarcts, WMH volume and decreased total white matter, grey matter and hippocampus volumes cross-sectionally and with a significant decline in only grey matter volume longitudinally.^131^ Conversely, other studies suggest that the diabetic status,^132^ duration of diabetes,^130,133^ glucose variability,^134^ or ethnic origin,^135^ but not the HbA1c level,^134^ are crucial in the development of cerebral microvascular lesions.

A pooled analysis of 3950 subjects living with diabetes from five population-based cohorts found that insulin use vs no insulin use was associated with increased risk of incident dementia and greater decline in cognitive function, which remained significant after adjustment for renal function and excluding persons whose diabetes was managed with lifestyle changes only, but not after using brain MRI measures.^136^ In the same analysis, there was no association between sulphonylurea use and incident dementia, but metformin use was associated with increased incident dementia in covariate-adjusted analyses.^136^ There were no associations of the three drugs with brain or WMH volumes.^136^ Multiple potential biases in these observational studies may explain the discrepant results (type of population, ethnicity, social context, education, age, duration of diabetes, type and duration of antidiabetic treatment, imaging measures, associated vascular risk factors).

Finally, there is some evidence from observational studies that diabetes or high levels of plasma glucose over extended periods are deleterious to the brain and may promote the development of SVD.^127^ There is also some evidence that long-term hyperglycaemia may promote cognitive decline in persons living with diabetes patients. Such effects may occur with the extension of cerebral SVD lesions, but also, independently, in connection with grey or white matter atrophy.^131^


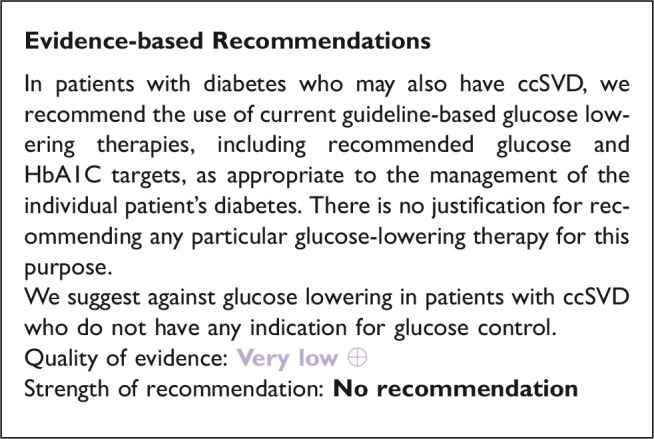


## Rational for expert consensus statement

The quality of available studies does not allow the guidelines writing group to provide any evidence-based recommendations for a specific glucose lowering therapy or for targeting a specific plasma glucose level or HbA1c in ccSVD patients with or without diabetes. There is also no evidence that a strict control of glycaemia may benefit patients with ccSVD who also have diabetes. There is no evidence to suggest that diabetes care guidelines^137^ should be altered in patients who also have ccSVD. Additional studies are warranted in persons living with diabetes with SVD to determine the ideal plasma glucose level below which extension of SVD lesions and clinical manifestations are prevented, whether specific glucose lowering treatments are more effective, and whether particular individual risk factors should be taken into consideration. Further prospective and randomised data are needed for this purpose.

Expert Consensus Statement

All group members agree that in prediabetes or diabetic patients with ccSVD:

• Glycaemic level should be appropriately monitored so as to be controlled according to the standards of medical care.

• We cannot advise any specific agent for obtaining appropriate glycaemic control.

• There is currently insufficient evidence to recommend targeting a specific glucose or HbA1c level distinct from the standard targets.

• There is no evidence to support any therapeutic intervention to reduce the normal glucose level.

**PICO 6:** In patients with covert cerebral small vessel diseases [WMH and/or lacunes], do conventional anti-dementia drugs [e.g. memantine, donepezil, galantamine, rivastigmine, etc.], compared to avoiding these interventions, reduce cognitive decline or dementia.

ccSVD carries an increased risk for development of cognitive complaints and cognitive impairment. Vascular cognitive impairment (VCI) captures all cognitive disorders related to cerebrovascular injury, from cortical ischaemic or haemorrhagic stroke to SVD.^43^ VCI ranges clinically from mild cognitive impairment, defined as impairment in at least one cognitive domain without clear influence on daily activities (Diagnostic and Statistical Manual of Mental Disorders, Fifth Edition, DSM-V, ‘minor neurocognitive disorder’), to fully developed dementia, defined as cognitive impairment in at least 2 cognitive domains which affects the person’s independent functioning (DSM-V ‘major neurocognitive disorder’). Dementia may be ‘pure’ vascular or mixed with degenerative Alzheimer’s disease. Contrary to the common statement that VCI due to SVD typically affects the cognitive domains of processing speed and executive functions, it affects any cognitive domain, including memory.^11,12^

Deficits in cholinergic and glutamatergic pathways are involved in cognitive decline in Alzheimer’s disease.^138,139^ There is also evidence for cholinergic involvement in VCI.^140^ Cholinesterase inhibitors increase acetylcholine levels by decreasing the rate at which it is broken down, thereby boosting cholinergic neurotransmission. Memantine is an NMDA receptor antagonist and protects against excessive glutamate-induced excitotoxicity which is hypothesized to be involved in the pathogenesis of Alzheimer’s disease, and also in vascular brain injury.

Based on these mechanisms of action in dementia, the working group defined a PICO question on whether these conventional anti-dementia drugs could reduce cognitive decline in patients with ccSVD. We searched for i) the cholinesterase inhibitors donepezil, galantamine and rivastigmine, ii) memantine, and iii) other anti-dementia drugs not covered in the other PICO questions.

## Analysis of current evidence

### Cholinesterase inhibitors

**Figure 8.**
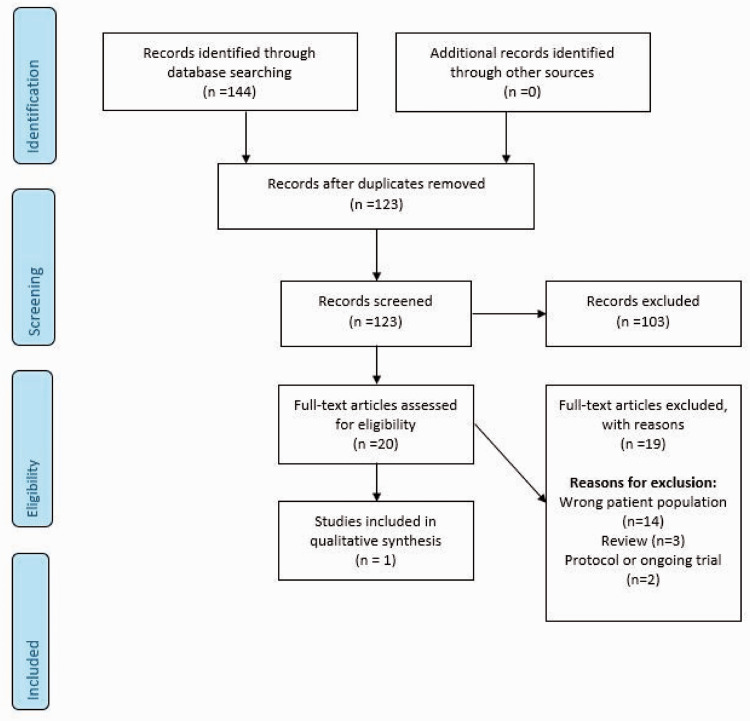
Summary of search results PICO 6.

We found no studies on donepezil, galantamine or rivastigmine meeting our criteria in ccSVD (Figure 8, Tables 11 and 12).

**Table 11.** Summary of clinical trial findings relevant for PICO 6.

Outcome	Study	Study design	Population	Age	Intervention	Comparison	Duration of follow-up	Intervention group		Control group		Summary of findings	RoB rating
								Number of events	Total in group	Number of events	Total in group		
Cognitive decline or Dementia	Bès 1999	RCT	Aged 60-82, HTN, leukoaraiosis on CT (>16 using Rezek method), no clinical signs of cognitive or neurological disorder	72 (Mean)	Nicergoline	Placebo	2 years	NA	36 (30 in intention-to-treat analysis)	NA	36 (31 in intention-to-treat analysis)	Significant difference in performance favouring Nicergoline found for memory performance on AVLT short term recall (mean difference: 3,6, p=0.026), AVLT delayed recall (mean difference: 1.7, p=0.013), Benton visual retention task (mean difference: 1.7, p=0.002); attention/concentration on Letter cancelation test (mean difference: 13.2, p=0.043), WAIS-R digit symbol sub-test (mean difference: 3.9, p=0.006); no significant difference found on 12 other tests.	High

RoB: risk of bias; RCT: randomised controlled trial; IPD: individual participant data; HTN: hypertension; AVLT: auditory verbal learning test; WAIS-R: Wechsler Adult Intelligence Scale-Revised; HTN: hypertension.

**Table 12.** Quality of results in trials relevant for PICO 6.

Certainty assessment							№ of patients		Effect		Certainty	Importance
№ of studies	Study design	Risk of bias	Inconsistency	Indirectness	Imprecision	Other considerations	Conventional dementia drugs	Control group	Relative (95% CI)	Absolute (95% CI)		
PICO 6.1 do conventional dementia drugs (I), compared to avoiding this intervention (C ) reduce risk of cognitive decline or dementia?												
1	Randomised trial	Very Serious	Not serious	Not serious	Very serious	None	30	31	Not pooled	Not pooled	⨁ Very Low	Critical

Notes: Downgraded 2 points high risk of bias; Downgraded 2 points for imprecision (very small sample size)

### Memantine

We found no studies on memantine in ccSVD.

### Other anti-dementia drugs

We found one RCT testing nicergoline, an ergot derivative with several mechanisms of action, i.e. it induces vasodilation and increases arterial blood flow, and enhances cholinergic and catecholaminergic neurotransmitter function. The study, performed 1990-1995, included 61 hypertensive patients with leukoaraiosis on brain CT and no clinical signs of cognitive or neurological disorders.^141^ Patients were randomly and double-blind assigned to nicergoline 60mg/day or placebo over a period of two years. Patients on nicergoline showed attenuated deterioration at the last visit (protocolled at two years, but unclear dropout) on three out of four memory tests, and two out of 10 attention and concentration tests. The effects on psychomotor and verbal function, and global cognitive performance, were non-significant. The quality of evidence, based on this single study with small sample size and multiple cognitive tests and uncertainty about dropout and a clinically relevant effect, was graded as very low because of serious risk of bias and very serious imprecision.

## Additional information

We believe there is both an under representation and under recognition of cognitive complaints and cognitive deficits in patients with SVD. Per definition, SVD is not ‘covert’ anymore when cognitive impairment, even mild, has been shown with formal cognitive testing. However, both (elderly) patients and their caregivers often do not notice or feel affected by mild and gradual cognitive decline, and also professionals often do not further explore cognition, even when vascular brain injury has been shown on imaging. We encourage more attention to cognitive impairment by actively asking for complaints, and performing cognitive screening by using appropriate tools.

### Cholinesterase inhibitors and memantine

Cholinesterase inhibitors and memantine are mainly indicated for treatment in Alzheimer’s disease. In VCI, several RCTs on these anti-dementia drugs have been performed. Most of these focused on vascular dementia and only few studies included mild VCI.

A recent comprehensive Cochrane review assessed the efficacy and safety of cholinesterase inhibitors in vascular dementia and other VCIs.^142^ The review included eight trials (4373 patients), three on donepezil, three on rivastigmine and two on galantamine. All trials included patients with vascular dementia, and one study also included post-stroke-cognitive impairment-no dementia. Donepezil and galantamine had small, unlikely to be clinically relevant effects, and rivastigmine had no important effect on cognition.

We mention for completeness one trial that tested 18 weeks of donepezil versus placebo in patients with mild cognitive impairment due to CADASIL.^143^ In 161 patients, there was no benefit of donepezil on the primary outcome (vascular AD assessment scale cognitive subscale) and small improvements in performance on Trails tests.

Another Cochrane review on memantine for dementia included three studies in vascular dementia.^144^ Mild VCI was not included. This review suggested that there is probably a small clinical benefit on cognitive function in vascular dementia for memantine compared with placebo. A post-hoc analysis indicated greater benefits in patients with more severe vascular dementia.

The Chinese clinical practice guideline for cognitive impairment of cerebral SVD, published in 2019, concluded there is level IIa evidence for the use of donepezil, galantamine and memantine in vascular dementia, but no recommendations were made for ccSVD or mild VCI.^145^

### Other anti-dementia drugs

Dl‐3‐n‐butylphthalidle (NBP), an anti‐VCI drug with multiple effects developed in China, was studied in a single, randomized, double‐blind, placebo‐controlled trial in 281 patients with VCI without dementia.^146^ Over a 6‐month treatment period, NBP was effective for improving cognitive and global functioning. This substance has not been studied in ccSVD.

Several substances that are available as dietary supplements, such as Ginkgo Biloba^147^ and CDP-choline or citicoline,^148,149^ have been studied in VCI and vascular dementia. Few studies were done in mild VCI, and none in ccSVD.

A Cochrane review (2009, not updated thereafter)^150^ on Ginkgo Biloba for cognitive impairment and dementia concluded that the evidence that Ginkgo Biloba has predictable and clinically significant benefit for people with dementia or cognitive impairment is inconsistent and unreliable. No subgroup analysis for (mild) VCI or vascular dementia could be done. A meta-analysis in 2015 concluded that Ginkgo Biloba is able to stabilize or slow decline in cognition in subjects with cognitive impairment and dementia, however there was no specific information on ccSVD, (mild) VCI or vascular dementia.^151^ In 2017, a small randomized, double-blind, placebo-controlled 6-month trial on Ginkgo Biloba in patients with VCI was published. However, VCI was unclearly defined as ‘cerebrovascular insufficiency’, and although there was a positive effect on the Clinical Global Impression scale, this was a per protocol analysis of only 58/90 patients who finished the study and it was concluded that further studies on efficacy and safety are still needed.^147^

The IDEALE study assessed the effect of a 9-month treatment with citicoline (500mg b.d.) in 349 patients with mild VCI, in a non-randomized open label setting. In the active treatment group, MMSE scores remained stable (22.4 at baseline and 22.9 at 9 months) while in the control group MMSE score declined 2 points (21.5 to 19.6 at 9 months).^149^

Other drugs, such as Cerebrolysin,^152^ huperzine^153^ and actovegin^154^ have been studied in vascular dementia or poststroke cognitive impairment, but not specifically in mild VCI nor in ccSVD.

We are not aware of any ongoing trials with (conventional) anti-dementia drugs such as are used in Alzheimer’s disease in ccSVD; few trials are ongoing in mild VCI aiming at reducing cognitive decline, such as a Chinese trial with i.m.-injections of mouse nerve growth factor in patients with VCI (https://clinicaltrials.gov/ct2/show/NCT04041349), and the CONIVaD trial,^155^ a RCT of nimodipine plus choline alphoscerate (a cholinergic precursor) or placebo during 12 months in patients with mild VCI due to SVD.


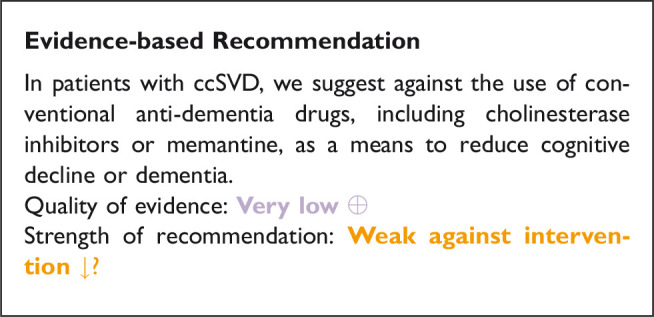


## Rational for expert consensus statement

These studies on conventional and other anti-dementia drugs in VCI and vascular dementia do not give much reason to presume any major effect on reduction of cognitive decline in mild VCI due to SVD or truly ccSVD, although it must be said that cognitive impairment in SVD develops slowly and the benefit may only show over many years, while the aforementioned trials only lasted 24 to 28 weeks. Moreover, the conventional anti-dementia drugs like cholinesterase inhibitors are symptomatic and do not act on the pathophysiological mechanism in SVD, so it is unlikely that they will be of any preventive benefit in truly covert cSVD. Trials in mild VCI and ccSVD are needed, with adequate duration of follow-up and appropriate neuropsychological testing covering the cognitive profile in VCI due to SVD. There is a gradual transition from truly ccSVD to cognitive complaints, cognitive impairment and dementia, and there is a need for a treatment guideline on VCI to fully cover this topic.

Expert consensus statement

Most group members suggest that: Considering the current lack of evidence for cholinesterase inhibitors and memantine in patients with ccSVD, and the small effects, at most, in patients with VCI or vascular dementia, we advice against prescribing these anti-dementia drugs in patients with ccSVD to prevent or reduce cognitive decline.

All group members suggest that: There is insufficient evidence for the use of any other anti-dementia drugs in patients with ccSVD to prevent or reduce cognitive decline.

## Discussion

Small Vessel Disease is a very common condition with substantial effects on individual subjects, their next-of kin, and the society as a whole. SVD can have many different clinical presentations, or can be covert (ccSVD). After considerable discussion of the SVD spectrum, the independent GRADE voting clearly identified ccSVD as the priority patient group (‘P’) for the current guideline, primarily because ccSVD is the commonest type of SVD and a very common challenge in clinical practice. Also, there is no current guideline on how to reduce clinical outcomes in ccSVD for the clinician, so clinical management may vary widely and be suboptimal and patients are concerned about what they should do.

*The* critical outcomes (‘O’) prioritised by independent voting of the Guideline Group to be essential to patients and clinical services in order of importance, were stroke, cognitive decline and dementia, dependency, mobility problems and mood disorders. In contrast, the Guideline Group placed the outcome of progression of imaging markers of SVD as too low a priority to include routinely in the current guideline because clinical outcomes are crucial to patients. Also, while imaging markers are very important as evidence of disease and often used in clinical trials, they bear an indirect relationship to clinical outcomes, and do not reflect the *whole patient* condition. In addition, it is difficult to uniformly assess SVD lesions or their change, the visible SVD features do not reflect the full extent of brain damage, sub-visible markers of SVD have limited acceptance, and the clinical implications of changes, such as one mL increase in WMH volume, are of less clinical value than those of having a stroke or receiving a diagnosis of dementia.

There are many potential interventions (‘I’), including those licenced for use in stroke or to manage vascular risk factors or other diseases but that could potentially be repurposed, as well as lifestyle modifying or dietary approaches. Many patients with ccSVD are treated with vascular risk factor modifying interventions, e.g. for hypertension, diabetes, or hyperlipidaemia, or antiplatelet therapies if they have ischaemic cardiovascular disease. Others receive treatments for dementias such as Alzheimer’s disease after visiting a memory clinic. Yet the impacts on ccSVD of these common guideline-based therapies for other conditions are unclear. The pathophysiology of SVD remains in debate and there may be several mechanisms of importance. The field of stroke has been dominated by decades of focus on atherosclerotic vessel occlusion, leading to ischaemia, how to stop the block and prevent ischaemia, so it is hardly surprising if these concepts occupy a prominent position in the mind when observing SVD lesions on a scan. And ‘vascular’ brain disease in memory or other non-stroke clinical presentations may lead back to the stroke-like thinking. So it is understandable if the reaction on noticing ccSVD on a brain scan is to consider aspirin treatment, or to assume that prescribing an antihypertensive drug will be beneficial. But are these treatments really justified, or could they be unsafe? The Guideline Group therefore prioritised to design a common guideline based on stroke prevention (antihypertensives, antiplatelets, lipid lowering drugs) and lifestyle interventions as important intervention priorities for evaluation, and also wanted to explore the role of glucose lowering agents (since diabetes is a major risk factor for SVD) and conventional dementia therapies (since many patients with SVD are seen in memory clinics).

Stroke presentations of SVD such as ‘lacunar’ stroke or ICH, are included within current regional or national stroke guidelines^85,86^ and situations with clinically clear manifestations of SVD were outside the scope of the current guideline. Monogenic forms of SVD were addressed recently.^9^ A previous guideline on silent cerebrovascular disease only considered stroke prevention and did not focus exclusively on ccSVD.^4^ A Chinese clinical practice guideline focussed on cognitive impairment in cerebral SVD.^145^ We also refer to current hypertension,^32^ diabetes,^137^ or dementia guidelines^145^ and white papers.^156,157^

We expected that there could be limited evidence on ccSVD and clinical outcomes. Not only was the amount of evidence limited, but the quality of evidence was generally low reflecting primarily the small sample sizes and methodologies including randomisation procedures. In view of our observed paucity of data, we also considered related evidence, such as from other likely-to-be-relevant clinical or asymptomatic populations, or effects on WMH studied with neuroimaging. We have focused on RCTs, systematic reviews and meta-analyses as much as possible, and include observational data only to a small extent. Even with this approach, the quality of evidence is very limited, although some of our recommendations are strong.

## Implications for clinical practice

Hypertension is a major risk factor for SVD and for stroke. Patients with hypertension should have their BP well controlled, with some evidence that lower BP targets may reduce WMH progression, supporting the use of lower suggested targets in cardiovascular disease prevention guidelines.^32^ However, there was no evidence to support the use of a specific antihypertensive drug class to reduce BP, or treatment with antihypertensive drugs in patients with ccSVD who do not have hypertension.

Patients with ccSVD should not be prescribed conventional antiplatelet drugs (most evidence exists for aspirin and clopidogrel), unless there is some other justifiable reason for doing so, such as ischaemic heart disease or after TIA/minor stroke, since conventional antiplatelet drugs do not appear to prevent stroke in patients without a history of symptomatic vascular disease. We found no evidence that conventional antiplatelet drugs prevent cognitive decline or dementia in subjects with ccSVD.^158^ Aspirin has not been shown to work for primary prevention of vascular disease^159^ and may be harmful if given long term as primary prevention, especially in elderly people (where there is a higher likelihood of ccSVD).^78^

We found that evidence on lipid lowering in ccSVD and clinical outcomes is very limited, in contrast to the considerable body of evidence on the benefits of lipid lowering in primary and secondary prevention of vascular disease,^99,100,102,160^ with limited evidence on WMH progression. However, the Guideline Group was somewhat divided on whether lipid lowering with statins should be considered in ccSVD.

We considered lifestyle interventions such as smoking cessation, exercise, dietary measures to avoid obesity and ensure good intake of vegetables, nuts and vitamins, good sleep habits, and avoidance of stress. Smoking cessation should be a priority for many reasons. We encourage aerobic exercise also for many reasons including some evidence of its beneficial effects on cognition. A healthy diet and avoidance of obesity are also strongly encouraged although this is for general health reasons rather than specifically because of evidence of benefit in ccSVD.^161^

We found no evidence on specific effects of glucose lowering regarding ccSVD; patients with type 2 diabetes should be managed according to glucose and HbA1c levels and interventions as advocated in diabetes management guidelines,^137^ with some evidence that good control of glucose levels may help reduce brain atrophy although it has no observed effect on WMH progression.

Although SVD is a common cause of cognitive impairment and dementia, including in mixed dementias, we found no evidence to support the use of conventional licenced anti-dementia treatments such as memantine or cholinesterase inhibitors in patients with only ccSVD, or indeed in vascular cognitive impairment.

## Implications for future research

Clinical management of ccSVD is hampered by difficulty recognising the atypical neurological, neuropsychiatric symptoms^10^ and cognitive profiles^11,12^ associated with higher burden of ccSVD lesions and likely to differentiate patients with a high risk of progression to clinical outcomes.^5^ Better recognition and definition of these symptoms might help identify patients at high risk of adverse SVD outcomes for focused clinical trials.

Research into SVD is also still hampered by varied terminology for SVD lesions^1^ and lack of agreed search strategies that capture the range of presentations and outcomes. Further efforts are needed to improve search standards for research into SVD.

Although the search identified 8302 references, the evidence-based management of ccSVD is severely hampered by a lack of direct evidence with many unanswered questions to address in future research (Table 13). Many large, observational cohort, other epidemiology, or genetic studies, have documented, beyond reasonable doubt, that common vascular disorders and lifestyle factors are clearly related to ccSVD. However, association is not causation. There are far too few trials testing whether modifying these factors reduces the important, debilitating, clinical effects of ccSVD, either individually or with combined modification approaches. The latter is important since risk factors commonly are related to each other and may have additive or multiplicative impacts. One reason for the limited impacts in ccSVD of e.g. BP lowering, on stroke or cognitive or WMH progression outcomes, may be because other risk factors remain being unaccounted for.

**Table 13.** Recommendations for future trials of ccSVD.

Variables	Details of variables	Comments
Description of SVD	MRI verified WMH, lacunes, microbleeds, perivascular spaces etc	MRI is preferable
Selection of subjects	No history/signs of previous stroke; VCI or dementia with selected type(s) of SVD from row above	Consider stratification by SVD lesion burden and lesion type
Size of trial	Based on adequate power calculations	Pilot study recommended before commencing larger trial
Description of baseline characteristics	Age, gender, ethnicity, educational level. If possible also socioeconomic status	
Vascular risk factors	Smoking, hypertension, BP levels, diabetes mellitus, blood glucose levels, atrial fibrillation, ischaemic heart disease, serum lipid levels	Co-variate adjustors or minimisation variables; also include age and ideally educational attainment or a measure of premorbid cognitive ability
Treatment	Double blind RCT is preferable	Or open label blinded outcome
Outcome measures	Ischaemic or haemorrhagic stroke, cognitive decline or dementia, dependency, death, major adverse cardiovascular events, mobility, or mood disorders	Primary outcome should be clinical; Secondary outcomes could include other clinical outcomes, and/or repeated MRI assessments of e.g. structural or vascular function measures
Observation period	At least 1 year, preferably longer; may depend on the outcome(s)	Follow-up at least every 6 months, including safety assessment

SVD: small vessel disease; MRI: magnetic resonance imaging; WMH: white matter hyperintensities; VCI: vascular cognitive impairment; BP: blood pressure; RCT: randomised controlled trial.

The lack of benefit of aspirin in ccSVD provides further evidence that SVD is not primarily atherothromboembolic, adding to evidence from genetic studies which have shown relations between WMH and hypertension but not atheroma genes^49^ and that the SVD stroke subtype was the only stroke subtype not associated with a genetic risk score for thrombosis.^162^

A non-atherothromboembolic SVD pathogenesis may appear to contradict the apparent benefit of cilostazol^73^ and potentially of statins.^21^ However, cilostazol is a weak antiplatelet drug with numerous other effects on the endothelium and white matter^163^ which could explain its apparent benefits in stroke prevention, particularly in small vessel stroke. Also, statins do not just lower lipids but have direct endothelial and anti-inflammatory effects which could benefit SVD. An obvious starting point for development of better drugs for SVD would be an exploration of these alternative modes of action of drugs like statins and cilostazol.

Furthermore, it is possible that atherothrombotic processes occur in damaged microvessels as an end stage of SVD lesion formation. Future studies should consider if effects of interventions differ between patients with different severities of ccSVD and with different proportions of WMH vs lacunes since these lesions may have different underlying pathologies.

ccSVD is considered as ‘asymptomatic’ but in fact is associated with subtle neurological as well as neuropsychiatric and cognitive symptoms^10^ which are associated with a high burden of lesions on brain imaging and therefore a high risk of stroke, dementia and other adverse outcomes,^5^ but are largely overlooked in clinics and probably by patients themselves. Better recognition and definition of the symptoms that *are* associated with ccSVD, as well as refined imaging and circulating biomarkers for ccSVD currently under investigation, may help identify a group of patients for ‘secondary prevention’, with a high burden of disease and higher risk for future adverse outcomes, who might benefit from aspirin or equivalent drugs, and this could be tested in clinical trials.

Numerous drugs, licenced for other purposes and therefore with known safety profiles, are available for testing and have relevant modes of action based on current knowledge of causes of sporadic SVD. There is increasing evidence that sporadic SVD is related to endothelial dysfunction, which affects myelin and inflammation as well as influencing brain fluid management, and is not primarily atherothrombotic. Drugs with multiple relevant modes of action of potential interest for ccSVD include cilostazol, dipyridamole, pentoxifylline, and various antihypertensive agents, all with presumed endothelial stabilising effects, some with myelin supporting effects, as well as statins and other anti-inflammatory drugs.^163^ Novel drug targets and therapeutic approaches that will enable specific treatments for the pathological processes underlying ccSVD are also needed, beyond the management of vascular risk factors.

Several trials have evaluated different interventions on SVD lesion progression on neuroimaging, mostly as part of secondary outcomes in a subset of trial participants, although most of them did not specifically target participants with ccSVD. While we strongly encourage future trials with clinical endpoints, collection of follow-up MRI would also be valuable to increase information on SVD lesion change and link this to clinical outcomes. But WMH progression is an unstandardized, and usually biased (due to typical losses to follow-up of at least 20%^54^), outcome measure that is of indirect relevance to clinical outcomes. There are also many trials evaluating other intermediary outcomes such as forearm blood flow, or cerebral blood flow, or diffusion tensor imaging, which are also not a substitute for hard clinical endpoints. Nor do such intermediary endpoint trials require smaller sample sizes than trials with hard clinical endpoints, since changes in WMH are typically small and fluctuations in WMH can have unexpected effects on sample size, including requiring substantial increases in sample.^164^

In contrast, there are few studies examining novel modifiable risk factors for ccSVD. Yet it is likely that there are many such risk factors beyond hypertension, diabetes, and atherosclerosis, since treatment of hypertension, even intensive treatment of hypertension, as discussed in PICO1, has such a limited effect on preventing SVD progression. There is a knowledge gap of understanding how additional mechanisms contribute to the development and deterioration of SVD. More research in these areas is urgently needed. Genomic and other high-throughput molecular approaches hold the promise of accelerating the discovery of mechanistic pathways underlying ccSVD, by providing new clues on biological pathways involved , with possibly applications for drug development and drug repositioning opportunities.

Trials with hard clinical endpoints targeting specifically patients with ccSVD are a priority going forward (Table 13). These trials should be powered to detect clinical outcomes. Analysis of epidemiological data to provide reliable estimates of clinical endpoints with credible confidence limits should be a priority. Since SVD can present in several different ways, trials should a) have broad entry criteria and b) carefully balance the randomisation for key prognostic variables including: SVD lesion burden; age; gender; mode of presentation; vascular risk factors particularly hypertension, smoking, diabetes; educational attainment and/or an estimate of premorbid cognitive ability, and possibly a measure of current socioeconomic status. Each of these are important predictors of baseline status and hence risk of disease progression, making it important to ensure that the trial groups are well balanced.

As more information about sporadic SVD mechanisms emerge, it may become important to consider trials in different SVD subtypes such as cerebral amyloid angiopathy, haemorrhagic forms of SVD, other clinical SVD entities, as well as subtypes of sporadic SVD selected for a high proportion of genetic risk variants.

Future guidelines should assess interventions for the common clinical presentations of SVD including lacunar ischaemic stroke, other types of ischaemic and haemorrhagic stroke with pre-existing SVD, MCI and dementia, mobility disorders and depression. These were outside the scope of the current guideline but account for a large number of persons attending stroke services, memory clinics, mobility and psychiatric clinics.

## Plain language summary

Covert (without any clear clinical symptoms) Small Vessel Disease is a common finding on brain imaging scans, especially in people 50-55 years or older, and is known to increase the future risk of stroke, dementia, dependency, mobility and mood disorders. There has been no current guideline to support the care of persons found to have ccSVD on brain scanning so as to reduce their risk of stroke, cognitive decline or dementia or other physical or mental problems that may restrict their independence. The ESO Guideline ccSVD Group was established and used approved methodologies to assess the current literature for evidence to help reduce the risk of health problems in persons found to have ccSVD.

We searched the literature up to December 2020 for studies on ccSVD and treatments to prevent stroke, cognitive decline or dementia, heart attacks, becoming dependent on someone else, problems with walking or mood and found a total of 8302 studies. We focused on commonly available treatments that are already used to prevent stroke such as aspirin, BP control, lowering the level of fats (cholesterol) in the blood, also lifestyle such as stopping smoking, exercise, diet including vitamins, sleep, and medications for making sure that the blood sugar is not too high and also for treating dementia. We focused on randomised clinical trials since they provide the most reliable evidence.

We found only a small number of studies to be about ccSVD but we used all the information that we considered to be relevant. This showed that patients with ccSVD should have their BP checked and if they have a high BP then they need to have their BP carefully controlled according to current BP guidelines. Unless they have a good reason, like a previous heart attack or stroke, patients with ccSVD should not take drugs like aspirin or clopidogrel to reduce the stickiness of platelets in the blood since these medications may not be beneficial for ccSVD. Patients with ccSVD can take statin treatment even if they have no other reason to do so since ccSVD represents a group of people with an increased risk of vascular events such as strokes and lipid lowering is beneficial in primary prevention of vascular disease – this is not likely to cause much harm and may do some good. Patients with ccSVD should not smoke – smoking is harmful in many ways including to the brain. They should take regular exercise since, in addition to the general health benefits of exercise, it may help to stop any decline in cognitive abilities and delay the onset of dementia. A healthy diet including plenty of green leafy vegetables, avoiding being overweight and good sleep habits are all important for general health. Patients with diabetes and ccSVD should focus on keeping their blood sugar well controlled according to current Diabetes Guidelines. We found no reason to suggest that drugs that are currently used to treat Alzheimer’s dementia such as cholinergic drugs or memantine, would help patients with ccSVD unless there was another reason for taking these drugs.

We emphasise and strongly encourage more research into ccSVD examining clinical outcomes that can be observed by the patients themselves or their relatives, and not just looking at whether the brain looks better on a scan. There are a wide range of other drugs that are currently licenced for other blood vessel or brain disorders or inflammatory disorders that have effects that need to be examined whether they might help stop the development and clinical diseases due to ccSVD. There is also a need for more novel drugs targeting mechanisms of action that are now being developed through research. All of these should be tested in clinical trials as soon as possible.

**Table:** 

Author	Intellectual and financial disclosures
Joanna M. Wardlaw	Intellectual disclosures: Grants from EU H2020, UK MRC, Fondation Leducq, Wellcome Trust, UK Stroke Association, British Heart Foundation and Alzheimer's Society for research into epidemiology, clinical and cognitive impacts and mechanisms of vascular dysfunction in small vessel disease. Chief Investigator of an ongoing randomised clinical trial (Lacunar Intervention trial 2, LACI-2) testing two repurposed drugs to prevent progression of small vessel disease. Financial disclosures: None
Hugues Chabriat	Intellectual disclosures: None Financial disclosures: Funded research: ANR(France) RHU TRT_cSVD
Charlotte Cordonnier	Intellectual disclosures: Chief Investigator of an ongoing randomised clinical trial (Avoid Anticoagulation after ICH, A3ICH) funded by the French Ministry of Health Financial disclosures: Speaker honorary of Boehringer Ingelheim Participation in Advisory Board of Biogen, Astra-Zeneca Participation in Executive committee for Trials Biogen, BMS.
Frank Erik de Leeuw	Intellectual disclosures: Clinical Established Investigator Dutch Heart Foundation. VIDI innovational award ZonMW. Financial disclosures: None
Stephanie Debette	Intellectual disclosures: Grants from EU H2020, JPND, ANR (French National funding agency, general and ‘Investment for the Future’ Programme ANR-18-RHUS-002, Claude Pompidou foundation, for research on environmental and genetic determinants of stroke, cerebral small vessel disease, MRI-markers of brain aging, cognitive decline and dementia PI on national investment for the future programme on covert cerebral small vessel disease (RHU SHIVA). Financial disclosures: None
Fergus Doubal	Intellectual disclosures: PI on LACI-2, RCT testing cilostazol and isosorbide mononitrate in lacunar stroke and PI on EU funded Horizon 2020 SVDs@TARGET trial of blood pressure lowering medication in lacunar stroke. Financial disclosures: Supported by fellowships from the Stroke Association Garfield Weston Foundation and NHS Research Scotland. Research grant funding from The Stroke Association, British Heart Foundation, Alzheimer’s Society
Christian Enzinger	Intellectual disclosures: None Financial disclosures: Speaker honorary of Biogen, Bayer, Celgene, Merck, Novartis, Roche, Shire, Genzyme, Teva Pharmaceutical Industries Ltd./Sanofi-Aventis. Participation in Advisory Boards: Bayer, Biogen, Celgene, Merck, Novartis, Roche and Teva Pharmaceutical Industries Ltd./sanofi-aventis.Funded research: Merck Serono, Biogen, and Teva Pharmaceutical Industries Ltd./Sanofi-Aventis
Hanna Jokinen	Intellectual disclosures: Grants from the Helsinki and Uusimaa Hospital District, University of Helsinki, and Päivikki and Sakari Sohlberg Foundation Financial disclosures: None
Arne G. Lindgren	Intellectual disclosures: National leader for Sweden and Denmark for the NAVIGATE study. National leader for Sweden for the PACIFIC Stroke study. Local PI for the StrokeCLOSE study and the Stat-ICH study. Financial disclosures: Funded research: The Swedish Research Council (2019-01757), NIH (1R01NS114045-01), The Swedish Government (under the “Avtal om Läkarutbildning och Medicinsk Forskning, ALF”), The Swedish Heart and Lung Foundation, Region Skåne, Lund University, Skåne University Hospital, Sparbanksstiftelsen Färs och Frosta, and the Fremasons Lodge of Instruction Eos in Lund. Personal fees from Bayer, Astra Zeneca, BMS Pfizer, and Portola.
Leonardo Pantoni	Intellectual disclosures: None Financial disclosures: None
Julie Staals	Intellectual disclosures: None Financial disclosures: Funded research: EU H2020 SVDs@target project; grant agreement No 666881, EU H2020 CRUCIAL project; grant agreement No 848109
Martin Taylor-Rowan	Intellectual disclosures: None Financial disclosures: None
Salvatore Rudilosso	Intellectual disclosures: None Financial disclosures: None
Sabrina Schilling	Intellectual disclosures: None Financial disclosures: None
Raffaele Ornello	Intellectual disclosures: None Financial disclosures: Speaker honoraria from Novartis, Teva, and Eli Lilly
Sebastian Eppinger	Intellectual disclosures: None Financial disclosures: None

## Supplemental Material

sj-pdf-1-eso-10.1177_23969873211012132 - Supplemental material for ESO Guideline on covert cerebral small vessel disease Click here for additional data file. Supplemental material, sj-pdf-1-eso-10.1177_23969873211012132 for ESO Guideline on covert cerebral small vessel disease by Joanna M Wardlaw, Stephanie Debette, Hanna Jokinen, Frank-Erik De Leeuw, Leonardo Pantoni, Hugues Chabriat, Julie Staals, Fergus Doubal, Salvatore Rudilosso, Sebastian Eppinger, Sabrina Schilling, Raffaele Ornello, Christian Enzinger, Charlotte Cordonnier, Martin Taylor-Rowan and Arne G Lindgren in European Stroke Journal
