# Supplementary material for: ESO Guideline on covert cerebral small vessel disease
Source: Eur Stroke J. 2021 May 11;6(2):CXI–CLXII. doi: 10.1177/23969873211012132 (PMC8370079; doi:10.1177/23969873211012132)
Supplement: sj-pdf-1-eso-10.1177_23969873211012132 - Supplemental material for ESO Guideline on covert cerebral small vessel disease [file sj-pdf-1-eso-10.1177_23969873211012132.pdf]

Search syntax

PICO 1 search:

1. ‘Cerebral small vessel disease*.mp.
2. White matter hyperintens*.mp.
3. white matter lesion*.mp.
4. white matter disease*.mp.
5. white matter change*.mp.
6. leukoaraiosis or leukoaraiosis/
7. cerebral small vessel disease.mp. or cerebrovascular disease/
8. MRI.mp. or nuclear magnetic resonance imaging/
9. computed tomography.mp. or computer assisted tomography/
10. OR/2-6
11. 6 OR 7
12. 10 AND 11
13. 8 OR 9
14. 12 AND 13
15. Lacun*.mp.
16. small deep infarct*.mp.
17. small subcortical infarct*.mp.
18. small deep stroke*.mp.
19. small subcortical stroke*.mp.
20. silent stroke*.mp.
21. silent brain infarct*.mp.
22. small vessel infarct*.mp
23. small vessel stroke*.mp.
24. old lacunar stroke.mp.
25. OR/15-24
26. brain/
27. cerebr*.mp.
28. lacunar stroke.mp.
29. cerebral small vessel disease.mp. or cerebrovascular disease/
30. OR/26-29
31. 25 AND 30
32. MRI.mp. or nuclear magnetic resonance imaging/
33. computed tomography.mp. or computer assisted tomography/
34. 32 OR 33
35. 31 AND 34
36. 1 OR 14 OR 35
37. *hypertension/dt
38. exp hypertension/dt
39. exp Antihypertensive Agents/
40. exp DIURETICS/
41. exp Ganglionic Blockers/
42. exp Adrenergic Antagonists/
43. exp Angiotensin-Converting Enzyme Inhibitors/

44. exp Calcium Channel Blockers/
45. exp Vasodilator Agents/
46. exp ADRENERGIC BETA-ANTAGONISTS/
47. exp ADRENERGIC ALPHA-ANTAGONISTS/
48. exp DIURETICS, THIAZIDE/
49. angiotensin II receptor antagonist$.tw.
50. exp LOSARTAN/
51. exp beta adrenergic receptor blocking agent/
52. OR/37-51
53. 36 AND 52

PICO2 search:

1. ‘Cerebral small vessel disease*
2. White matter hyperintens*
3. white matter lesion*
4. white matter disease*
5. white matter change*
6. leukoaraiosis or leukoaraiosis/
7. cerebral small vessel disease.mp. or cerebrovascular disease/
8. MRI.mp. or nuclear magnetic resonance imaging/
9. computed tomography.mp. or computer assisted tomography/
10. OR/2-6
11. 6 OR 7
12. 10 AND 11
13. 8 OR 9
14. 12 AND 13
15. Lacun*
16. small deep infarct*
17. small subcortical infarct*
18. small deep stroke*
19. small subcortical stroke*.mp.
20. silent stroke*.mp.
21. silent brain infarct*.mp.
22. small vessel infarct*.mp
23. small vessel stroke*.mp.
24. old lacunar stroke.mp.
25. OR/15-24
26. brain/
27. cerebr*.mp.
28. lacunar stroke.mp.
29. cerebral small vessel disease.mp. or cerebrovascular disease/
30. OR/26-29
31. 25 AND 30
32. MRI.mp. or nuclear magnetic resonance imaging/
33. computed tomography.mp. or computer assisted tomography/
34. 32 OR 33
35. 31 AND 34
36. 1 OR 14 OR 35
37. exp antithrombocytic agent/
38. (antiplatelet$ or anti-platelet$ or (platelet$ adj5 inhibit$) or (thrombocyt$ adj5 inhibit$)).tw.
39. (alprostadil$ or aspirin$ or dipyridamol$ or disintegrin$ or epoprostenol$ or iloprost$ or ketanserin$ or ketorolac tromethamine$ or milrinone$ or mopidamol$ or pentoxifyllin$ or ticlopidine$ or thiophen$ or trapidil$ or prasugrel or terutroban).tw,tn.
40. (acetyl salicylic acid$ or acetyl?salicylic acid or clopidogrel$ or picotamide$ or ligustrazine$ or levamisol$ or suloctidil$ or ozagrel$ or oky046 or oky-046 or defibrotide$ or cilostazol or satigrel or sarpolgrelate or kbt3022 or kbt-3022 or isbogrel or cv4151 or cv-4151 or triflusal).tw,tn.

41. (Dispril or Albyl$ or Ticlid$ or Persantin$ or Plavix or Aggrenox or Pletal).tw,tn.
42. exp fibrinogen receptor/
43. (((glycoprotein iib$ or gp iib$) adj5 (antagonist$ or inhibitor$)) or GR144053 or GR-144053 or abciximab$ or tirofiban$ or eftifibatid$).tw.
44. (ReoPro or Integrilin$ or Aggrastat).tw,tn.
45. exp thrombocyte activation/
46. exp thrombocyte/
47. (sulphinpyrazone or sulfinpyrazone or indobufen).tw.
48. Or/37-47
49. 36 AND 48

PICO 3 search:

1. ‘Cerebral small vessel disease*
2. White matter hyperintens*
3. white matter lesion*
4. white matter disease*
5. white matter change*
6. leukoaraiosis or leukoaraiosis/
7. cerebral small vessel disease.mp. or cerebrovascular disease/
8. MRI.mp. or nuclear magnetic resonance imaging/
9. computed tomography.mp. or computer assisted tomography/
10. OR/2-6
11. 6 OR 7
12. 10 AND 11
13. 8 OR 9
14. 12 AND 13
15. Lacun*
16. small deep infarct*
17. small subcortical infarct*
18. small deep stroke*
19. small subcortical stroke*.mp.
20. silent stroke*.mp.
21. silent brain infarct*.mp.
22. small vessel infarct*.mp
23. small vessel stroke*.mp.
24. old lacunar stroke.mp.
25. OR/15-24
26. brain/
27. cerebr*.mp.
28. lacunar stroke.mp.
29. cerebral small vessel disease.mp. or cerebrovascular disease/
30. OR/26-29
31. 25 AND 30
32. MRI.mp. or nuclear magnetic resonance imaging/
33. computed tomography.mp. or computer assisted tomography/
34. 32 OR 33
35. 31 AND 34
36. 1 OR 14 OR 35
37. exp hyperlipidemia/
38. exp antilipemic agents/
39. hypercholesterol$.tw.
40. hyperlipid$.tw.
41. statin$.tw.
42. antilipid$.tw.
43. hyperlip?emia.tw.
44. dyslip?emia.tw.
45. lipid lowering.tw.

46. PCSK9 inhibitors
47. Ezetimibe
48. OR/37-47
49. 36 AND 48

PICO 4 search:

1. ‘Cerebral small vessel disease*
2. White matter hyperintens*
3. white matter lesion*
4. white matter disease*
5. white matter change*
6. leukoaraiosis or leukoaraiosis/
7. cerebral small vessel disease.mp. or cerebrovascular disease/
8. MRI.mp. or nuclear magnetic resonance imaging/
9. computed tomography.mp. or computer assisted tomography/
10. OR/2-6
11. 6 OR 7
12. 10 AND 11
13. 8 OR 9
14. 12 AND 13
15. Lacun*
16. small deep infarct*
17. small subcortical infarct*
18. small deep stroke*
19. small subcortical stroke*.mp.
20. silent stroke*.mp.
21. silent brain infarct*.mp.
22. small vessel infarct*.mp
23. small vessel stroke*.mp.
24. old lacunar stroke.mp.
25. OR/15-24
26. brain/
27. cerebr*.mp.
28. lacunar stroke.mp.
29. cerebral small vessel disease.mp. or cerebrovascular disease/
30. OR/26-29
31. 25 AND 30
32. MRI.mp. or nuclear magnetic resonance imaging/
33. computed tomography.mp. or computer assisted tomography/
34. 32 OR 33
35. 31 AND 34
36. 1 OR 14 OR 35
37. Exercise/ or Exercise Therapy/
38. Physical Exertion/
39. Motor Activity/
40. Sports/
41. sport$.tw.
42. exp "Physical Education and Training"/
43. (physical adj3 (activit$ or education$ or exertion$ or training)).tw.
44. exercise$.tw.
45. exp diet therapy/

46. ((diet or dieting) adj5 (health$ or weight$)).tw.
47. (calorie adj3 (control or reduc$ or restriction)).tw.
48. food choice$.tw.
49. (fat camp$ or weight loss camp$).tw.
50. nutrition education.tw.
51. Nutrition Therapy/
52. behavior therapy/
53. Cognitive Therapy/
54. psychotherapy/
55. (behavio?r$ adj3 (therap$ or technique$ or modif$ or intervention$)).tw.
56. (cognit$ adj3 (therap$ or technique$ or modif$ or intervention$)).tw.
57. CBT.tw.
58. (psychotherap$ or psycho-therap$).tw.
59. family therapy/
60. (family adj3 (therap$ or intervention$)).tw.
61. family-based.tw.
62. sedentary lifestyle/
63. (sedentary adj3 (lifestyle or behavio?r$)).tw.
64. (psycho-social or psychosocial).tw.
65. exp Health Promotion/
66. Health Education/
67. (health$ adj3 (promot$ or educat$ or lifestyle)).tw.
68. lifestyle/
69. (lifestyle$ or life-style$).tw.
70. smoking/
71. Sleep/
72. CPAP/
73. community*based intervention
74. community*based programme
75. exp diet/
76. Diet, Mediterranean/
77. (mediterranean adj3 diet*).tw.
78. (mediterranean adj6 food*).tw.
79. (mediterranean adj6 nutrition*).tw.
80. (mediterranean adj6 eat*).tw.
81. (salt adj3 (increase* or reduce* or decrease*)).mp
82. (sugar adj3 (increase* or reduce* or decrease*)).mp.
83. exp fruit
84. exp vegetables
85. smoking cessation.mp.
86. exp smoking cessation/ or smoking cessation program/
87. exp smoking
88. 53 and (((quit$ or stop$ or ceas$ or giv$ or prevent$) adj3 smok$) or cigarette$).ti,ab.
89. exp smoking habit/
90. smoking reduction/
91. nicotine adj3 (substitute or patch)
92. exp *vitamin/
93. exp diet supplementation/

94. vitamin D/
95. vitamin B complex/ or vitamin B group/
96. vitamin D/
97. vitamin K epoxide reductase/ or vitamin K group/
98. vitamin supplementation/
99. "folic acid".ti,ab.
100. folic acid/
101. "vitamin* supple*".ti,ab.
102. OR/37-101
103. 36 AND 102

PICO 5 search:

1. ‘Cerebral small vessel disease*
2. White matter hyperintens*
3. white matter lesion*
4. white matter disease*
5. white matter change*
6. leukoaraiosis or leukoaraiosis/
7. cerebral small vessel disease.mp. or cerebrovascular disease/
8. MRI.mp. or nuclear magnetic resonance imaging/
9. computed tomography.mp. or computer assisted tomography/
10. OR/2-6
11. 6 OR 7
12. 10 AND 11
13. 8 OR 9
14. 12 AND 13
15. Lacun*
16. small deep infarct*
17. small subcortical infarct*
18. small deep stroke*
19. small subcortical stroke*.mp.
20. silent stroke*.mp.
21. silent brain infarct*.mp.
22. small vessel infarct*.mp
23. small vessel stroke*.mp.
24. old lacunar stroke.mp.
25. OR/15-24
26. brain/
27. cerebr*.mp.
28. lacunar stroke.mp.
29. cerebral small vessel disease.mp. or cerebrovascular disease/
30. OR/26-29
31. 25 AND 30
32. MRI.mp. or nuclear magnetic resonance imaging/
33. computed tomography.mp. or computer assisted tomography/
34. 32 OR 33
35. 31 AND 34
36. 1 OR 14 OR 35
37. exp antidiabetic agent/
38. (glucose lowering and (therap$ or agent$ or drug$)).tw.
39. (hypoglycemic and (agent$ or drug$ or therap$)).tw.
40. (antidiabet$ and (agent$ or drug$ or therap$)).tw.
41. metformin.tw.
42. rosiglitazone/
43. rivoglitazone/

44. pioglitazone/
45. troglitazone/
46. exp dipeptidyl peptidase IV inhibitor/
47. glucagon-like peptide-1.tw.
48. incretin mimetic$.tw.
49. exp alpha glucosidase inhibitor/
50. sodium glucose cotransporter 2/
51. Sodium glucose co-transporter 2 inhibitor$.tw.
52. exp glucagon like peptide/
53. sglt2 inhibitor
54. Sulfonylurea
55. *glitazon*
56. Insulin
57. Diabetic diet
58. OR/37-57
59. 36 AND 58

PICO 6 search:

1. ‘Cerebral small vessel disease*
2. White matter hyperintens*
3. white matter lesion*
4. white matter disease*
5. white matter change*
6. leukoaraiosis or leukoaraiosis/
7. cerebral small vessel disease.mp. or cerebrovascular disease/
8. MRI.mp. or nuclear magnetic resonance imaging/
9. computed tomography.mp. or computer assisted tomography/
10. OR/2-6
11. 6 OR 7
12. 10 AND 11
13. 8 OR 9
14. 12 AND 13
15. Lacun*
16. small deep infarct*
17. small subcortical infarct*
18. small deep stroke*
19. small subcortical stroke*.mp.
20. silent stroke*.mp.
21. silent brain infarct*.mp.
22. small vessel infarct*.mp
23. small vessel stroke*.mp.
24. old lacunar stroke.mp.
25. OR/15-24
26. brain/
27. cerebr*.mp.
28. lacunar stroke.mp.
29. cerebral small vessel disease.mp. or cerebrovascular disease/
30. OR/26-29
31. 25 AND 30
32. MRI.mp. or nuclear magnetic resonance imaging/
33. computed tomography.mp. or computer assisted tomography/
34. 32 OR 33
35. 31 AND 34
36. 1 OR 14 OR 35
37. exp Cholinesterase Inhibitors/
38. ("acetylcholinesterase inhibitor*" or "cholinesterase inhibitor*" or "anti-cholinesteras*").mp.
39. donepezil*.mp.
40. aricept*.mp.
41. donezepil.mp.
42. exp *Galantamine/
43. galantamin*.ti,ab.
44. galanthamin*.mp.

45. Nivalin*.mp.
46. Razadyne*.mp.
47. Reminyl*.mp.
48. rivastigmin*.mp.
49. exelon*.mp.
50. exp *Tacrine/
51. tacrin*.ab,ti.
52. cognex*.mp.
53. ("anti-dementia drug*" or "memory drug" or "anti-alzheimer* ADJ2 drug*").mp.
54. exp *Memantine/
55. memantin*.mp.
56. (axura* or akatinol*).mp.
57. namenda*.mp.
58. (ebixa* or abixa*).mp.
59. memox*.mp.
60. OR/37-59
61. 36 AND 60
